# Physicochemical and Rheological Characterization of Ferrimagnetic Polymer–Ceramic Colloidal Gels as Potential Inks for Direct Ink Writing

**DOI:** 10.3390/ma19214496

**Published:** 2026-10-26

**Authors:** Ankita Raja Shinde, Vasudev Vivekanand Nayak, Jingyang Wang, Marcus Weck, Nikhil Gupta, Lukasz Witek

**Affiliations:** 1 Biomaterials and Regenerative Biology Division, NYU College of Dentistry, New York, NY 10010, USA; axs10184@nyu.edu; 2 Department of Mechanical and Aerospace Engineering, NYU Tandon School of Engineering, Brooklyn, NY 11201, USA; 3 Department of Biochemistry and Molecular Biology, University of Miami Miller School of Medicine, Miami, FL 33136, USA; 4 Dr. John T. Macdonald Biomedical Nanotechnology Institute (BioNIUM), University of Miami, Miami, FL 33136, USA; 5 Molecular Design Institute, Department of Chemistry, New York University, New York, NY 10012, USAmw125@nyu.edu (M.W.); 6 Department of Biomedical Engineering, NYU Tandon School of Engineering, Brooklyn, NY 11201, USA; 7 Hansjörg Wyss Department of Plastic Surgery, NYU Grossman School of Medicine, New York, NY 10016, USA; 8 Department of Oral and Maxillofacial Surgery, NYU College of Dentistry, New York, NY 10010, USA

**Keywords:** direct inkjet writing, bone, scaffolds, tissue regeneration, rheology

## Abstract

Direct ink writing (DIW) enables patient-specific fabrication of multifunctional bone scaffolds, but formulating inks that combine ceramic osteoconductivity, magnetic hyperthermia capability, and controlled drug delivery while retaining printability remains challenging. This study investigates the composition-dependent rheology and printability of polycaprolactone (PCL)/β-tricalcium phosphate (β-TCP)/magnetite (Fe_3_O_4_) colloidal gels (formulated inks) for DIW-based scaffold fabrication. Five formulations spanning 10–50 wt% PCL (with β-TCP adjusted accordingly and Fe_3_O_4_ fixed at 5 wt%) were synthesized via solvent-based gel casting, characterized, and evaluated rheologically (flow sweep, amplitude and frequency sweeps, three-interval thixotropy, gelation kinetics). All inks exhibited shear-thinning behavior. Intermediate PCL compositions showed higher storage moduli, broader linear viscoelastic regions, higher yield stress, and rapid post-shear recovery, indicative of well-percolated particle–polymer networks, while high-ceramic-loaded formulations displayed brittle behavior. Calculated yield pressures for intermediate compositions fell within practical DIW operating ranges. Printing tests confirmed that only intermediate solid loadings are reliably printed through both nozzle diameters, demonstrating that printability depends jointly on yield stress and solid fraction. These findings establish composition-based guidelines for optimizing multifunctional PCL/β-TCP/Fe_3_O_4_ inks toward printable, patient-specific bone scaffolds.

## 1. Introduction

Direct ink writing (DIW), also known as robocasting, is an extrusion-based additive manufacturing process that utilizes a feedstock of colloidal suspensions (inks). The DIW process involves three distinct, interconnected sub-functions, each with specific rheological requirements for the ink. During extrusion, the ink experiences elevated shear rates (typically >10 s^−1^) as it flows through the nozzle under an applied pressure [1,2]. The ink then subsequently transitions from high-shear to low-shear conditions during the solidification phase, which directly follows its deposition onto a substrate [1,2]. This is followed by the final process, where the deposited ink exhibits the ability to form spanning lattices by rapidly attaining the desired structural properties to withstand compressive stresses [3]. This is typically achieved through rheological optimization, with the ink displaying shear-thinning behavior, appropriate yield stress, viscoelastic properties, and rapid recovery characteristics [1,3].

DIW offers the unique advantage of printing at room temperature [4]. This aspect is particularly crucial for polymer-rich inks, where excessive heating could potentially alter viscosity and accelerate binder decomposition. Temperature control is also important for cell-laden bioinks, as processing conditions help preserve cell viability while maintaining the shear-thinning and yield-stress behavior necessary for extrusion and shape fidelity [5]. Among the inks used in DIW, calcium phosphate ceramics, such as β-tricalcium phosphate (β-TCP) and hydroxyapatite, play a crucial role in tissue regeneration and have demonstrated excellent biocompatibility, osteo-conductivity, and the ability to support new bone formation in preclinical and clinical studies [6,7]. In malignant bone tumors, for example, surgical resection of the lesion often results in critical-size defects, where these bioceramic scaffolds can provide both structural support and a favorable microenvironment for the regeneration of form and function at the defect site [8].

DIW enables these materials to be organized into interconnected porous architectures in which filament dimensions, pore geometry, and scaffold architecture can be controlled through ink formulation and printing parameters [9,10]. However, highly ceramic-loaded ink formulations can be difficult to extrude and may exhibit limited structural stability or brittle behavior after processing. Incorporation of polymers into ceramic-containing inks therefore provides a strategy for modifying their rheological behavior, processability, and mechanical characteristics [11]. Polycaprolactone (PCL) is particularly attractive for composite scaffold fabrication because of its biodegradability, biocompatibility, processability, and established use in biomedical and drug-delivery applications [12]. PCL/calcium phosphate composite scaffolds have consequently been investigated using extrusion-based additive manufacturing to combine the processability of the polymer phase with the osteoconductive characteristics of calcium phosphate ceramics [13]. Beyond conventional binary polymer–ceramic systems, recent developments in additive manufacturing have increasingly focused on multifunctional scaffolds in which additional phases are incorporated to impart therapeutic or stimulus-responsive properties [13,14]. Magnetic iron oxide (Fe_3_O_4_) particles are of interest in this context because they can provide magnetic responsiveness and can generate heat when exposed to an alternating magnetic field [8,15]. Magnetic hyperthermia has been investigated as a localized therapeutic approach, and Fe_3_O_4_-mediated magnetic hyperthermia has also reached clinical application for selected indications, demonstrating the broader translational potential of magnetically responsive materials [15]. Incorporating a magnetic phase into a polymer–ceramic scaffold therefore provides a potential route toward constructs that combine structural functionality with externally activated thermal response.

Previous studies have demonstrated the suitability of DIW and related robocasting approaches for fabricating bone-repair scaffolds using the individual material classes incorporated in the present system. For the polymeric component, Zhang et al. fabricated PCL and PCL/polyethylene oxide (PEO) woodpile constructs by DIW and demonstrated that solvent composition and PEO incorporation influenced ink rheology, surface characteristics, and wettability, highlighting the feasibility of processing PCL-based biomaterials by DIW at room temperature [16]. For the ceramic component, Miranda et al. developed concentrated β-TCP inks with suitable viscoelastic properties for robocasting scaffolds with controlled three-dimensional (3D) geometry and mesoscale porosity, demonstrating the importance of ink formulation for successful extrusion and structural retention [17]. For magnetic functionality, Zhang et al. fabricated 3D-printed β-TCP bioceramic scaffolds modified with an Fe_3_O_4_/graphene oxide nanocomposite interface, where the incorporation of the magnetic phase enabled magnetothermal heating for potential hyperthermia treatment of bone tumors [18].

Collectively, these studies demonstrate the feasibility of using PCL-based polymers, β-TCP ceramics, and Fe_3_O_4_-containing magnetic systems in additively manufactured biomaterial constructs. However, integrating polymeric, ceramic, and magnetic phases into a single highly filled DIW ink introduces additional rheological complexity because variations in composition can influence particle–polymer interactions, flow resistance, yield behavior, viscoelasticity, and structural recovery after extrusion. Despite increasing interest in multifunctional scaffolds, the effect of systematically varying the polymer-to-ceramic ratio within a ternary PCL/β-TCP/Fe_3_O_4_ colloidal-gel system on its rheological behavior and DIW processability remains insufficiently characterized.

Previous work has demonstrated the individual feasibility of PCL-based DIW, β-TCP robocasting, and Fe_3_O_4_-containing magnetic scaffolds; this study systematically examines how changing the PCL/β-TCP ratio at fixed Fe_3_O_4_ content governs the rheological behavior and DIW processability of the combined ternary system. The specific contribution of this work is the establishment of composition-dependent relationships between the polymer–ceramic ratio, rheological behavior, and DIW processability of this ternary PCL/β-TCP/Fe_3_O_4_ system. Rather than demonstrating biological performance, the present study provides a materials-processing and rheological foundation for the reproducible fabrication of multifunctional composite scaffolds.

## 2. Materials and Methods

### 2.1. Materials

#### 2.1.1. Iron Oxide Preparation Materials

Hydrochloric acid (HCl, ≥36%) was purchased from Sinopharm Chemical Reagent Co., Ltd., Shanghai, China. Sodium hydroxide (NaOH, 96%), iron(iii) chloride hexahydrate (FeCl_3_·6H_2_O, 99%), and iron(ii) chloride tetrahydrate (FeCl_2_·4H_2_O, 99%) were obtained from Aibi Chemistry Preparation Co., Ltd., Shanghai, China. Chloroform stabilized with amylene (Bdh, product no. Bdh1109-4lg) was purchased from VWR, Radnor, PA, USA. All chemicals were used as received without further purification unless otherwise specified.

#### 2.1.2. Formulated Ink Materials

Commercially available PCL [manufacturer-specified average number average molecular weight (Mn) = 80,000 g/mol; polydispersity index < 2; product no. 440744] was purchased from Sigma-Aldrich, Burlington, MA, USA. Calcium phosphate tribasic, (Ca_3_(PO_4_)_2_; 34–40% Ca; Mn 310.17 g/mol; Cas No. 12167-74-7) was used as the ceramic component and purchased from Thermo Fisher Scientific, Waltham, MA, USA.

### 2.2. Preparation and Processing of Raw Materials

Fe_3_O_4_ particles were synthesized using a co-precipitation method adapted from previous work by Zhang et al. (Figure S1, see the Supplementary Materials) [19]. Producing Fe_3_O_4_ particles in the same controlled setting as the subsequent scaffold fabrication step helped material traceability, which is critical for the regulatory approval of an invasive device [20]. In addition, this in-house synthesis was chosen due to cost-effectiveness at larger scales [21]. In brief, 5.2 g of FeCl_3_·6H_2_O, 3.825 g of FeCl_2_·4H_2_O, and 0.85 mL of concentrated HCl (≥36%) were dissolved in 25 mL of deionized water. A 250 mL NaOH solution (1.5 M) was then added dropwise to the mixture under vigorous stirring, resulting in a microparticle formation, maintaining a reagent ratio of approximately FeCl_3_·6H_2_O: FeCl_2_·4H_2_O: HCl: NaOH of 1: 1:0.5:5. The resulting supernatant was discarded, and the precipitate was washed with 100 mL of deionized water while being centrifuged for 10 min at 2000 rpm. This washing and centrifugation process was repeated 3 times. Subsequently, the precipitate was dried in a vacuum oven (Model VD23, Binder GmbH, Tuttlingen, Germany) at 60 °C and 100 mbar for 24 h, and Fe_3_O_4_ particles (<37 µm diameter) were sieved. Separately, β-TCP powder was calcined at 800 °C for 11 h and the powder was attrition milled (Union Process, Akron, OH, USA) for 60 min in ultrapure water using 1.0 mm diameter zirconia milling media. Following milling, the ceramic suspension was separated from the media, and the resulting slurry was dried in a low-temperature oven (~80 °C) for ~12 h or until the complete evaporation of water was observed.

### 2.3. Raw Material Characterization

Raw materials were analyzed by powder X-ray diffraction (XRD) using a diffractometer (X’Pert Powder, Panalytical, Almelo, The Netherlands) to verify the crystalline phases. The diffraction data were collected over a 2θ range of 20–80° with a step size of 0.013°, at an operating voltage of 45 kV, and current of 40 mA. The resulting spectra were evaluated using a proprietary software package (Highscore analytical, Panalytical, Almelo, The Netherlands). Fourier transform infrared (FTIR) spectra of the samples were acquired using a spectrometer (PerkinElmer, Waltham, MA, USA) over a wavenumber range of 4000–400 cm^−1^ at a spectral resolution of 4 cm^−1^. Spectra were acquired in transmittance mode and reported as percent transmittance (%T), with KBr used as the background.

Scanning electron microscopy (SEM) was used to characterize the surface topography of the Fe_3_O_4_ and β-TCP powders. Powders were imaged using a field-emission SEM (Zeiss Gemini 300, Oberkochen, Germany) operated at 10 kV with a field width of 400 μm. Energy-dispersive X-ray spectroscopy (EDS) was performed using backscattered secondary electron detector (Zeiss Gemini 300, Oberkochen, Germany) to verify the elemental composition. To prevent charging, samples were coated with a 10 nm layer of carbon using a carbon coater (CCU-010 Safematic GmbH, St. Gallen, Switzerland). For EDS, a working distance of ~6.3 mm was chosen at 10 kV and 1.1 nA, and 3 samples were analyzed per group. To assess the molecular-weight distribution of PCL, gel permeation chromatography (GPC) was employed using a liquid chromatograph (LC-20AT, Shimadzu Corporation, Kyoto, Japan) coupled with an autosampler (SIL-20A HT, Shimadzu Corporation, Kyoto, Japan). The analysis was conducted in tetrahydrofuran (THF), with polystyrene standards serving as calibration references. Specimens were prepared at a concentration of 3 mg/mL, and measurements were performed at a controlled flow rate of 1.0 mL/min. For GPC testing, ePCL comprising 25% PCL + chloroform (solvent) was analyzed against control PCL pellets (cPCL).

The particle-size distribution of the β-TCP powder was quantified from 5 SEM micrographs using automated image analysis implemented in Python (version: 3.13.16) within a Google Colab cloud-computing environment (utilizing google-colab package version: 0.0.1a2). Each micrograph was calibrated using its corresponding SEM spatial calibration (µm/pixel). Images were converted to grayscale, smoothed using a Gaussian filter, and contrast-enhanced using contrast-limited adaptive histogram equalization (CLAHE), followed by Otsu thresholding to distinguish particulate features from the background. Small, segmented regions attributable to image noise or thresholding artifacts were removed using a minimum projected-area criterion. A minimum projected area of 3.47 µm^2^, corresponding to a 20-pixel area in the reference micrograph (0.4166 µm/pixel), was used as the common physical-area criterion. Because the micrographs had different spatial calibrations, this physical-area threshold was converted to the corresponding pixel area for each image to maintain a consistent exclusion criterion across all micrographs. Touching particulate features were separated using a distance-transform watershed algorithm. A minimum peak separation of 7 pixels was selected following the visual assessment of the segmentation overlays to provide the appropriate separation of the adjacent features while limiting artificial over-segmentation. Objects intersecting the image boundaries were excluded. The projected area of each segmented feature was converted to an equivalent circular diameter (ECD), ECD = 2√A/Π, where (A) is the projected area. A total of 5756 segmented features from 5 SEM fields were included in the number-based particle-size distribution.

### 2.4. Formulated Ink Synthesis

The formulated ink approach described by Zhang et al. [19] was modified for use in DIW (Figure S2). The gels were prepared using commercially available PCL (Sigma-Aldrich, Burlington, MA, USA). This molecular weight range was chosen to ensure adequate polymer chain entanglement for structural integrity while maintaining solubility in chloroform for gel preparation. PCL was dissolved in 20 mL of chloroform under constant agitation at room temperature. β-TCP and Fe_3_O_4_ particles were then added to the solution. Five distinct gel compositions (as detailed in Table 1) were chosen to investigate the impact of varying PCL and β-TCP ratios on rheological properties and printability of the composite colloidal gel. A constant Fe_3_O_4_ concentration of 5 wt% was maintained across all formulations to isolate the effects of the polymer–ceramic ratio. At such loading concentrations, a previous study reported cytocompatibility of Fe_3_O_4_ containing composites [22]. Using 5 wt% Fe_3_O_4_ also ensures that the local iron dose is relatively low and thereby mitigates the risk of oxidative stress-mediated toxicity [22]. The PCL concentrations, ranging from 10 wt% to 50 wt%, were selected to span a broad spectrum from ceramic-dominant to polymer-dominant inks, relevant for DIW applications. The individual mixtures were stirred in a sealed container for 30 min until complete dissolution was achieved.

### 2.5. Rheological Characterization of the Colloidal Gels

The formulated inks were analyzed for their rheological properties using a rotational rheometer (Discovery Hybrid Rheometer 2, TA Instruments, New Castle, DE, USA) at 23 °C. Measurements were conducted between parallel plates with a diameter of 20 mm, separated by a 1 mm gap. The shear rate was varied logarithmically from 0.001 s^−1^ to 1000 s^−1^ over 100 s. Frequency sweep tests were conducted by varying the oscillation frequency logarithmically from 0.1 to 100 Hz. Amplitude sweeps were also performed by varying the strain logarithmically from 1% to 100% at a constant frequency of 1 Hz. Moreover, 3-interval thixotropy tests (3ITT, or recovery tests) were performed in 3 successive stages by applying strain amplitudes in the following sequence: first, a low strain of 1% (100 s) was applied, followed by a high strain of either 100% or 500% (100 s), and then a return to 1% strain (300 s). This protocol enabled the assessment of the sample’s ability to recover its mechanical properties after being subjected to large deformations.

To calculate the yield pressure (ΔP_y_), which represents the minimum extrusion pressure required to initiate the flow through the nozzle, the force balance in laminar capillary flow was calculated as the wall shear stress is related to the applied pressure drop as follows:

(1) $$\tau_{w}=\frac{\mathrm{PR}}{2L}$$

where the pressure drop across the nozzle (P), wall shear stress (τ_w_) equals the material yield stress (τ_y_, obtained from oscillatory or steady shear rheology), signifying the onset of yielding of the polymer–ceramic network. Below this pressure, the material is assumed to behave as a viscoelastic solid; and above it, a yielded annulus forms adjacent to the nozzle wall, thereby enabling steady flow.

At this threshold (τ_w_ = τ_y_), the corresponding yield pressure is obtained as follows:

(2) $$\Delta P_{y}=\frac{2L\tau_{y}}{R}$$

where L is the nozzle length (12.7 mm) and R is the nozzle radius (0.255 mm or 0.205 mm). These values are standard tip diameters obtained directly from the manufacturer (Nordson EFD, Westlake, OH, USA).

This formulation assumes an axisymmetric cylindrical flow (no slip at the wall) and provides the first-order criterion for the extrusion onset. Above ΔP_y_, the plug radius (r_o_) decreases progressively according to the following equation:

(3) $$r_{o}=R\times \frac{\tau_{y}}{\tau_{w}}$$

Equation (3) illustrates the transition from solid-like plug behavior (r < r_o_) to the sheared annular region (r_o_ < r < R), where r is an arbitrary radius.

The yield–pressure calculation provides a first-order estimate of the pressure required to initiate flow through the deposition nozzle. A related rheology-based approach was reported by Fatimi et al. for injectable calcium phosphate suspensions, in which high-shear flow behavior was fitted using the Ostwald-de Waele power-law model and the injection pressure was predicted from the flow properties, needle geometry, and volumetric flow rate [23]. Their findings demonstrated that the pressure required for extrusion is governed not only by the rheological properties of the suspension but also by the needle radius, needle length, and imposed flow rate. In addition, agreement between the calculated and measured injection pressures was obtained when the needle diameter was sufficiently large relative to the particle size. For narrower needles, particle depletion and possible wall slip caused the experimentally measured injection pressure to be lower than the theoretical prediction [23].

Although the present study concerns DIW with PCL/β-TCP/Fe_3_O_4_ colloidal gels rather than the syringe injection of calcium phosphate pastes, the same general principle applies, where rheological behavior and nozzle geometry jointly influence the pressure required for material extrusion. Therefore, the calculated yield pressure in the present study should be interpreted as a comparative estimate of extrusion onset under the assumptions of laminar, axisymmetric capillary flow and no wall slip. The actual printing pressure may additionally depend on flow rate, high shear viscosity, particle–wall interactions, and possible slip or particle segregation within the nozzle.

### 2.6. Printing Parameters

The formulated inks were transferred into a syringe (Nordson, Westlake, OH, USA) fitted with tip diameters of 0.51 mm and 0.41 mm (Nordson, Westlake, OH, USA). Scaffold fabrication was carried out using a custom-designed DIW 3D printer (3D Inks LLC, Tulsa, OK, USA) following a design process implemented in RoboCAD v5 (3D Inks LLC, Tulsa, OK, USA). Printing was conducted using an orthogonal strand pattern to achieve a lattice-like configuration. The print speed was set at 8 mm/s for all ink formulations at room temperature (25 °C). The inks were extruded onto a dry substrate, and the printed scaffolds were subsequently dried under ambient conditions. Final drying was performed in an low-temperature oven at 30 °C for 24 h.

### 2.7. Scanning Electron Microscope of Printed Scaffolds

The surface morphology and pore/strand architecture of the printed scaffolds were examined using a table-top scanning electron microscope (SEM; Hitachi High-Tech Corporation, TM4000, Tokyo, Japan). Following fabrication, the scaffolds were mounted on aluminum SEM stubs using conductive carbon tape and imaged without sputter coating. Micrographs were acquired from the top surface of the scaffolds in secondary electron (SE) imaging mode at an accelerating voltage of 10 kV. Representative micrographs were obtained at 200× magnification for P10B85F5, P20B75F5, P30B65F5, P40B55F5, P50B45F5, and PCL scaffolds.

### 2.8. XRD and FTIR of Printed Scaffolds

The phase composition and crystallinity of the scaffolds were analyzed by XRD. Scaffolds from all formulation groups were tested. Prior to analysis, samples were finely grounded to obtain homogeneous powders. XRD measurements were performed using an X’Pert Powder diffractometer (PANalytical, Almelo, The Netherlands) operated at 45 kV and 40 mA. Diffraction patterns were collected over a 2θ range of 20–80° with a step size of 0.020°. The resulting diffractograms were examined to identify characteristic peaks corresponding to β-TCP, PCL, and Fe_3_O_4_, enabling assessment of crystalline phase distribution within the composite scaffolds [24,25,26].

FTIR spectroscopy was used to identify functional groups and assess potential chemical interactions among scaffold components. Printed scaffolds were ground into fine powder, and approximately 1 mg of each sample was mixed homogeneously with 25 mg of spectroscopic-grade KBr. The mixtures were pressed into pellets using a hydraulic press at 5 MPa for 1 min. FTIR spectra were recorded in the range 4000–400 cm^−1^ using the on a spectrometer (PerkinElmer, Waltham, MA, USA). A pure KBr pellet prepared under identical conditions served as the background reference to minimize atmospheric and instrumental interference [27,28].

### 2.9. Statistical Analysis

Data obtained from the rheological (gelation) tests were analyzed using one-way ANOVA, where *p* < 0.05 was considered statistically significant. SPSS (v31, IBM, Armonk, NY, USA) was used for statistical analysis.

## 3. Results

### 3.1. Raw Material Characterization

#### 3.1.1. XRD

**Figure 1:**
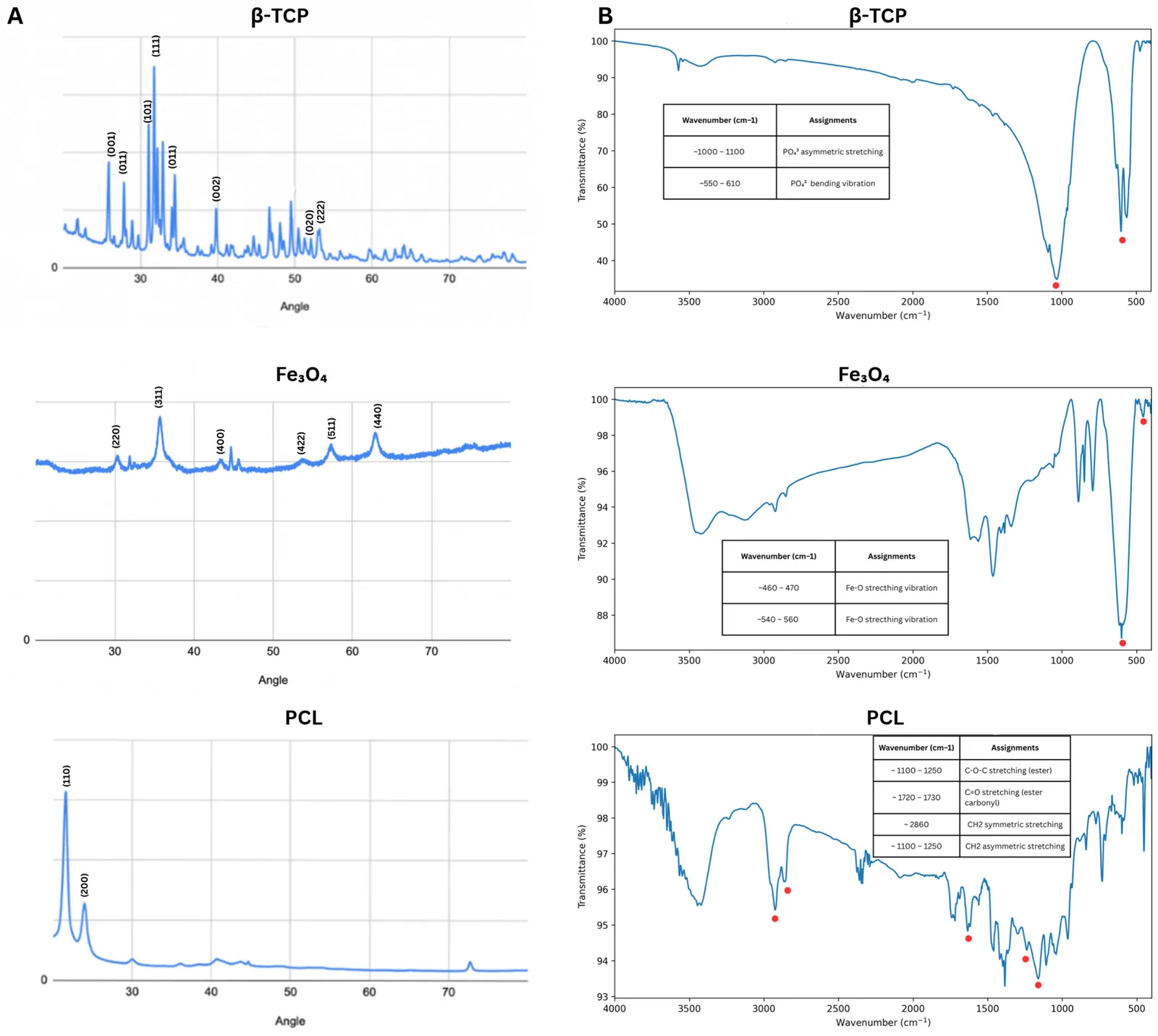
(**A**) XRD, with intensity shown in arbitrary units (a.u.), and (**B**) FTIR spectra of the raw materials used for scaffold fabrication. Red dots highlight the major peaks on the FTIR spectra.

The characteristic β-TCP peaks (Figure 1A) appeared at 2θ values of approximately 25.9°, 27.8°, 31.0°, 34.4°, and 35.8°, with additional peaks observed at approximately 39.8°, 52.1°, and 53.0°, closely matching reported β-TCP crystallographic data [29,30,31]. In contrast, PCL exhibited broad and less intense peaks observed at approximately 21.3° and 23.6° [32,33]. Fe_3_O_4_ (magnetite) exhibited characteristic peaks at approximately 30.1°, 35.5°, 43.1°, 53.4°, 57.0°, and 62.6°, corresponding to the (220), (311), (400), (422), (511), and (440) crystallographic planes, respectively [34]. Although diffraction features below 20° were outside the experimentally acquired range, multiple characteristic Fe_3_O_4_ peaks within the measured range supported the phase assignment as magnetite [24].

#### 3.1.2. FTIR

FTIR spectrum (Figure 1B) for Fe_3_O_4_ was characterized by strong Fe-O vibrations in the 400–700 cm^−1^ region, confirming the Fe_3_O_4_ structure [35]. The absorption band at ~560 cm^−1^ corresponds to the intrinsic metal–oxygen (Fe–O) bond in the inverse spinel structure. Additional weaker spectral features observed near ~630 cm^−1^ further substantiate the formation of crystalline magnetite. PCL spectra showed a prominent ester C=O stretching band near 1720 cm^−1^, distinct aliphatic C–H_2_ stretching vibrations at 2945 and 2865 cm^−1^, and C–O–C stretching vibrations between 1160 and 1180 cm^−1^ [36].

#### 3.1.3. SEM and EDS

**Figure 2:**
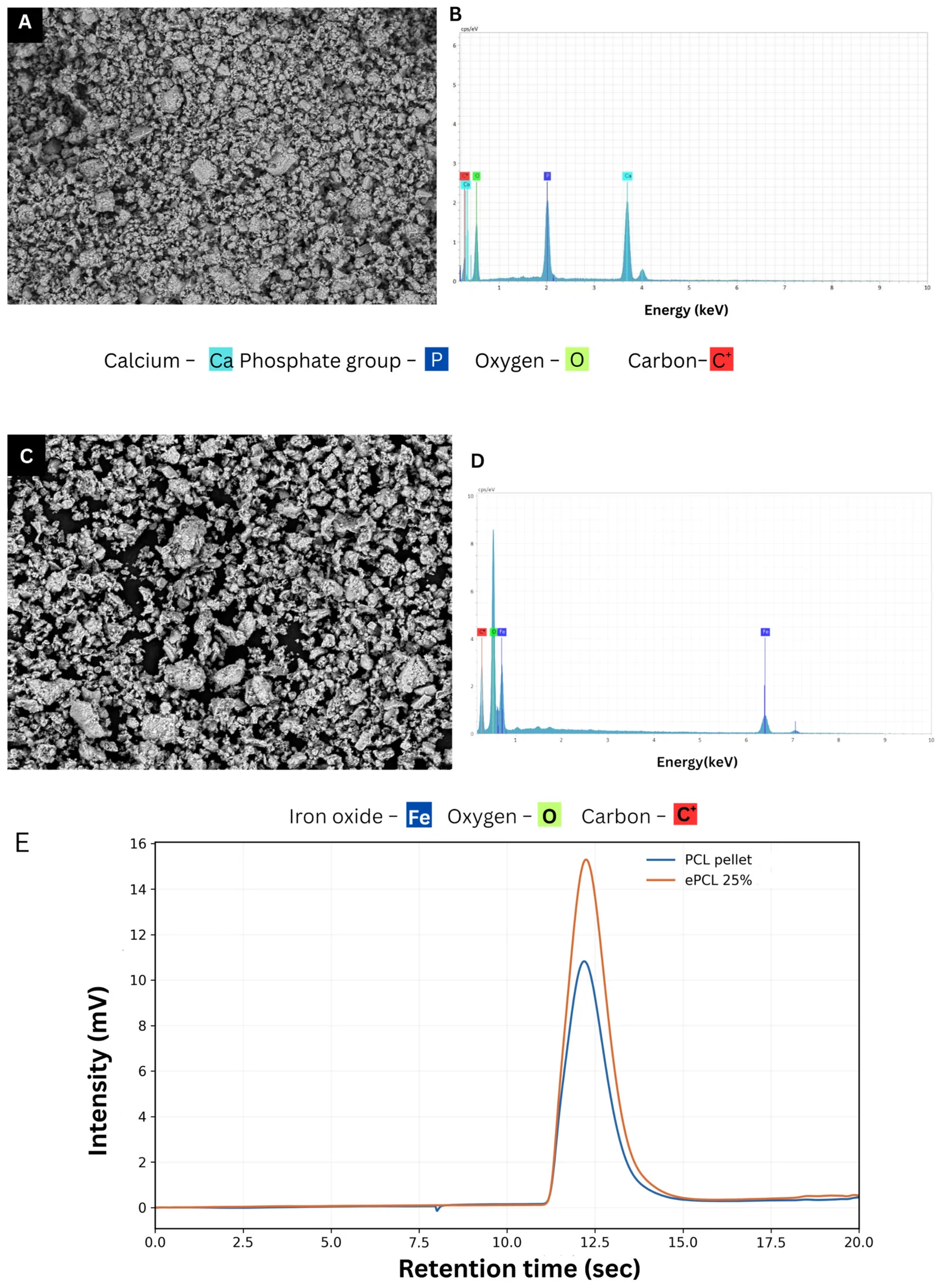
(**A**) SEM of β-TCP powder, and (**B**) corresponding EDS spectrum revealing the elemental composition, with prominent peaks for calcium (Ca), phosphorus (P), and oxygen (O). (**C**) SEM micrograph of Fe_3_O_4_ particles and (**D**) corresponding EDS spectrum revealing the elemental composition, with strong peaks for iron (Fe) and O. (**E**) GPC profiles of cPCL and ePCL.

The SEM micrograph of the β-TCP powder (Figure 2A) revealed a heterogeneous morphology comprising fine, irregularly shaped granules with agglomeration. The EDS spectrum (Figure 2B) confirmed the elemental composition of β-TCP, showing prominent peaks corresponding to calcium (Ca) at 3.7 keV, phosphorus (P) at 2 keV, and oxygen (O) at 0.5 keV, the key constituents of the Ca_3_(PO_4_)_2_ phase [37]. SEM micrograph of the Fe_3_O_4_ particles (Figure 2C) displayed a dense and granular morphology. EDS analysis (Figure 2D) exhibited characteristic peaks of iron (Fe) and O, near 6.4 keV and 0.5 keV, respectively, confirming the oxide composition [38]. The absence of extraneous peaks indicates high phase purity and the absence of contamination during co-precipitation or drying. The presence of a carbon (C) peak could be attributed to the carbon coating.

#### 3.1.4. GPC

GPC was performed on the cPCL and ePCL to assess changes in molecular-weight distribution associated with processing. The samples exhibited substantially overlapping principal elution peaks, with the ePCL peak appearing sightly later than that of the cPCL (Figure 2E). The ePCL sample exhibited lower Mn and Mw values of the 131,572 and 199,932 g/mol, respectively, compared with 137,477 and 208,804 g/mol for the cPCL. Dispersity remained nearly unchanged, with values of 1.5196 for ePCL and 1.5189 for the cPCL [39].

#### 3.1.5. Particle Analysis

The pooled number-based ECD distribution comprised 5781 segmented features, with a mean ECD of 6.12 ± 3.09 µm and a median (P50) of 5.46 µm (Figure S3). The P10 and P90 values were 2.94 and 10.05 µm, respectively, indicating that 80% of the measured particulate features had ECD values between approximately 3.0 and 10.5 µm. The observed ECD range was 2.11–33.14 µm.

### 3.2. Rheological Analysis

#### 3.2.1. Flow Sweep

**Figure 3:**
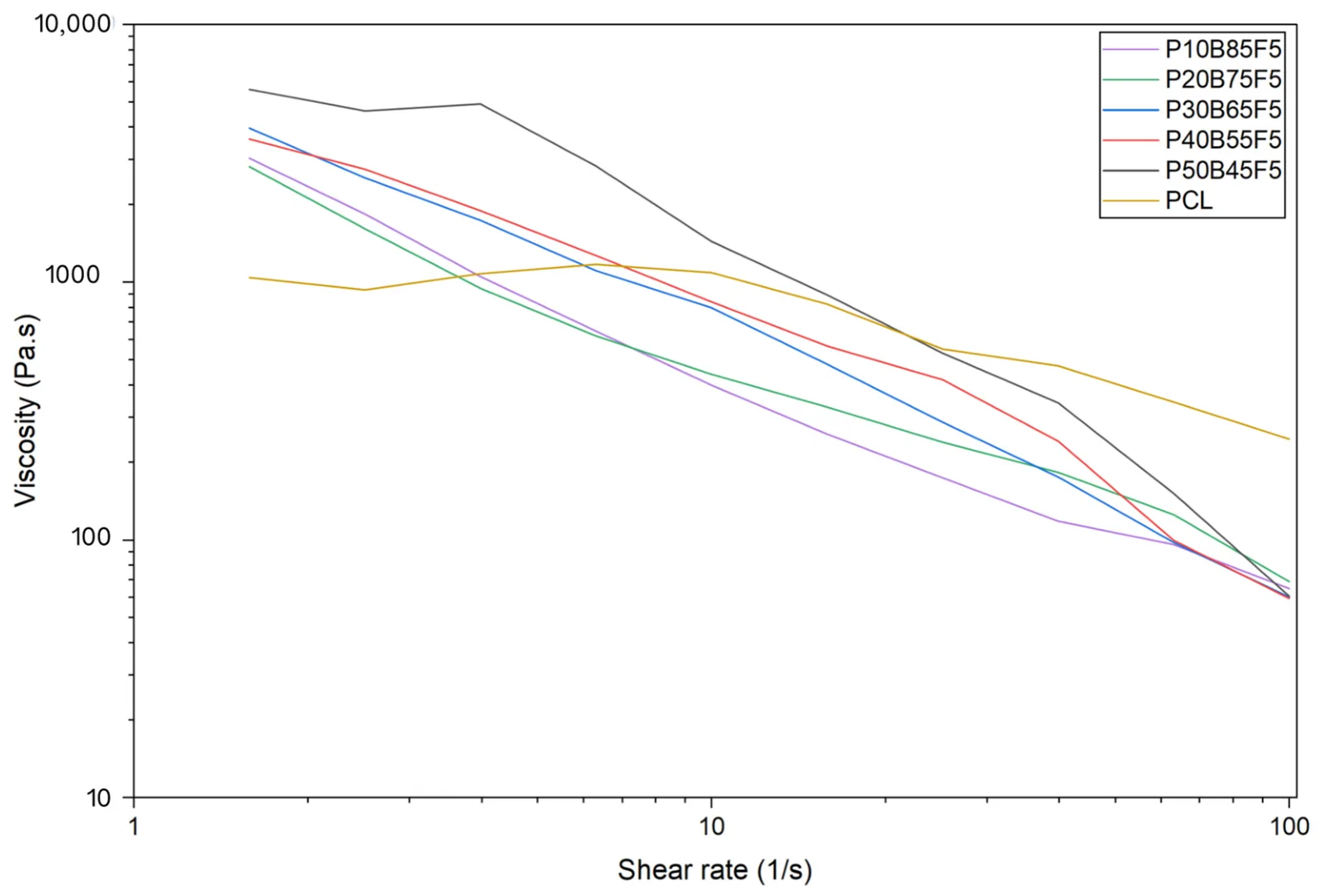
Shear-thinning plot of all compositions with data presented as means for all formulated ink compositions (P10B85F5, P20B75F5, P30B65F5, P40B55F5, P50B45F5, and PCL).

Flow sweep measurements showed increasing the shear-rate-reduced the viscosity for all experimental groups (Figure 3). The results revealed shear-thinning characteristics across all solvent gel-cast groups, with viscosity increasing as the ceramic concentration in the gel increased.

#### 3.2.2. Amplitude Sweep

The variation in the storage modulus (G′) with oscillation stress for all composite formulations (P10B85F5–P50B45F5) is shown in Figure S4. An amplitude sweep test was performed to determine the linear viscoelastic region (LVR), the range over which the material exhibits a constant modulus independent of applied stress, and to identify the onset of structural breakdown or yielding. The resulting data revealed transitions from linear to nonlinear viscoelastic behavior (Figure S5).

At low oscillation stresses, all samples exhibited a plateau in G′, indicating that the composite responded elastically. Within this region, the material behaved like a viscoelastic solid. As the applied stress increased beyond a critical point, G′ began to decrease markedly, signifying the end of the LVR and the onset of network disruption or yielding behavior. Among the formulations, P30B65F5 exhibited the highest initial G′ values (~10^4^–10^5^ Pa) within the LVR, followed closely by P40B55F5 and P20B75F5, indicating a percolated microstructure with strong particle–matrix interactions and stress transfer. In contrast, P50B45F5 showed the lowest G′ throughout the entire stress range, reflecting a weaker, more polymer-dominant network with limited filler connectivity. P10B85F5 maintained a high storage modulus at low stress but began to decline earlier than P30B65F5, suggesting an earlier onset of structural breakdown, possibly due to brittle agglomerate networks caused by excessive ceramic content. The yield point, defined as the stress at which G′ starts to deviate from its plateau value, increased with polymer content up to P30B65F5, beyond which it decreased. This indicates that a moderate polymer content promoted better particle wetting and cohesive reinforcement, whereas excessive polymer reduces the rigidity of the network. The broader LVR observed for P20B75F5, P30B65F5, and P40B55F5 demonstrated greater structural stability under oscillatory loading, an essential property for maintaining filament integrity during extrusion-based printing.

#### 3.2.3. Frequency Sweep

**Figure 4:**
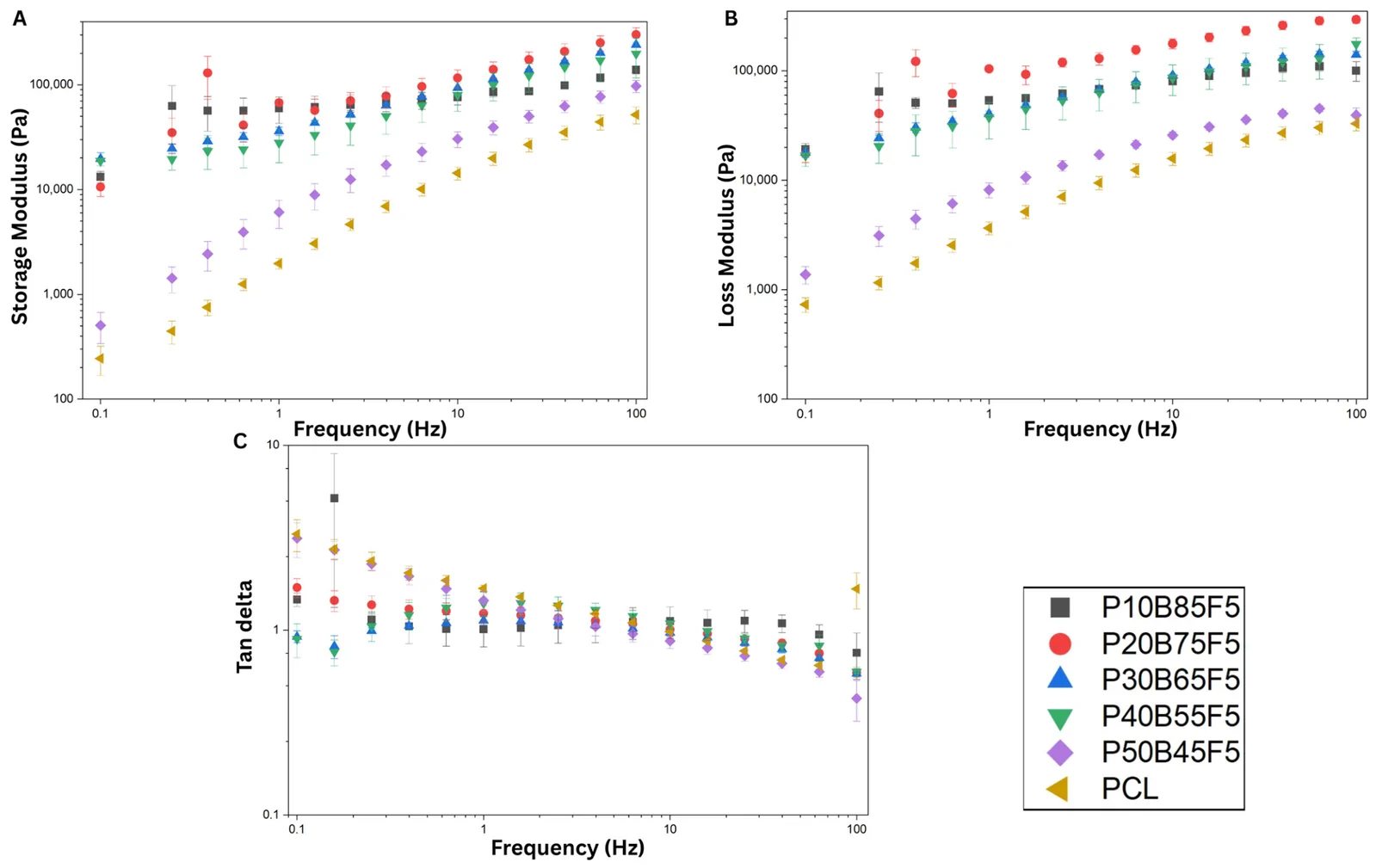
Frequency sweep rheological behavior of composite scaffold inks. (**A**) Storage modulus (G′), (**B**) loss modulus (G″), and (**C**) tan δ as a function of frequency. Data are presented as mean ± standard error.

Frequency sweep analysis revealed a pronounced dependence of both the G′ (Figure 4A) and loss modulus (G″) (Figure 4B) on the oscillation frequency for all composite formulations. In general, both moduli increased with increasing frequency, reflecting the viscoelastic nature of the inks. At low frequencies, the materials exhibited lower resistance to deformation. However, as the frequency increased, the gel responded elastically, leading to increased energy storage and dissipation. Among all formulations, P20B75F5 consistently exhibited the highest values of both G′ and G″ throughout the frequency range. P30B65F5 displayed comparable behavior. In contrast, P50B45F5 showed lower moduli, implying a transition toward a more polymer-dominant, compliant structure with reduced filler reinforcement and higher chain mobility. Increasing PCL content initially enhances the moduli up to a critical ratio (P20B75F5–P30B65F5) and then diminishes at higher polymer fractions.

The variation in tan δ (G″/G′) with frequency for all PCL/β-TCP/Fe_3_O_4_ composite formulations is presented in Figure 4C. The tan δ profile demonstrated that increasing frequency shifted the behavior from viscous- to elastic-dominated in all composite formulations. At very low frequencies, all samples showed elevated tan δ values; P10B85F5 and P50B45F5 exceeded values of 2–5. This indicated that these materials are highly viscous and less elastic at low frequencies. For all samples, tan δ first rapidly decreased with increasing frequency (<1 Hz), plateaued (between 1–10 Hz), and then further decreased (between 10–100 Hz). P20B75F5, P30B65F5, and P40B55F5 demonstrated intermediate tan δ values at both low and high frequencies (generally around or just below 1 at high frequency). In summary, these tan δ results align with modulus data and supports the interpretation that intermediate compositions yield more reliable viscoelastic performances suitable for 3D-printing applications.

#### 3.2.4. Yield Stress and Viscoelastic Response

The yield stress increased progressively from P10B85F5 to P40B55F5, reaching a maximum of ~1.33 kPa (Figure S5). The yield stress of P50B45F5 was much lower than any other group which was ~477 Pa. The rheological properties (G’ and G″) did not change linearly, but rather peaked at intermediate composition, showcasing similar non-monotonic behavior. P30B65F5 and P40B55F5 exhibited the highest moduli. Across all groups except P10B85F5, G″ remained greater than G′. The relatively high G′/G″ ratio observed for P30B65F5 reflects improved elastic energy storage and structural recovery after deformation; these are attributes that favor dimensional stability and minimize filament (extruded formulated ink) spreading during extrusion. The yield strain (γ_γ_) increased progressively from 2.8% for P20B75F5 to 6.5% for P50B45F5, signifying a transition from rigid, particle-dominated rheological behavior to more polymer-dominated rheological behavior. Compositions exhibiting moderate oscillatory yield stress (900–1200 Pa) and moderate yield strain (3–4%), specifically P30B65F5 and P40B55F5, provided a balance between flowability under shear and shape recovery post-deposition (Table 2).

ΔP_y_ were calculated for each composite formulation (P10B85F5–P50B45F5) using the experimentally measured τ_γ_. The results, presented in Table 2, represent the minimum onset pressure required to initiate flow in a 0.51 mm or 0.41 mm diameter, and 12.7 mm length precision deposition tip. These values indicate that higher β-TCP loading (P40B55F5) leads to a stronger particle–polymer network, requiring higher pressures for initiating extrusion [40]. However, ΔP_γ_ decreased sharply for P50B45F5 (47.5 kPa). The calculated yield pressures were within the typical operational range of DIW systems (50–150 kPa).

Further, one-way ANOVA revealed significant differences among the formulations in storage modulus (F (5,12) = 12.014, *p* < 0.001), loss modulus (F (5,12) = 18.496, *p* < 0.0001), and oscillation stress (F (5,12) = 8.878, *p* = 0.001), whereas no significant difference was observed for oscillation strain (F (5,12) = 2.716, *p* = 0.073), as shown in Table S1. Tukey’s HSD post hoc analysis was subsequently performed for pairwise comparisons, with the complete results provided in Table S2. (Note: ΔP_γ_ is simply yield stress multiplied by a constant. Therefore, ANOVA on ΔP_γ_ produces essentially the same F statistic and *p*-value as the yield-stress ANOVA. Hence, the we have considered yield stress, storage modulus, loss modulus and oscillation strain (%) for analysis.)

#### 3.2.5. Recovery

**Figure 5:**
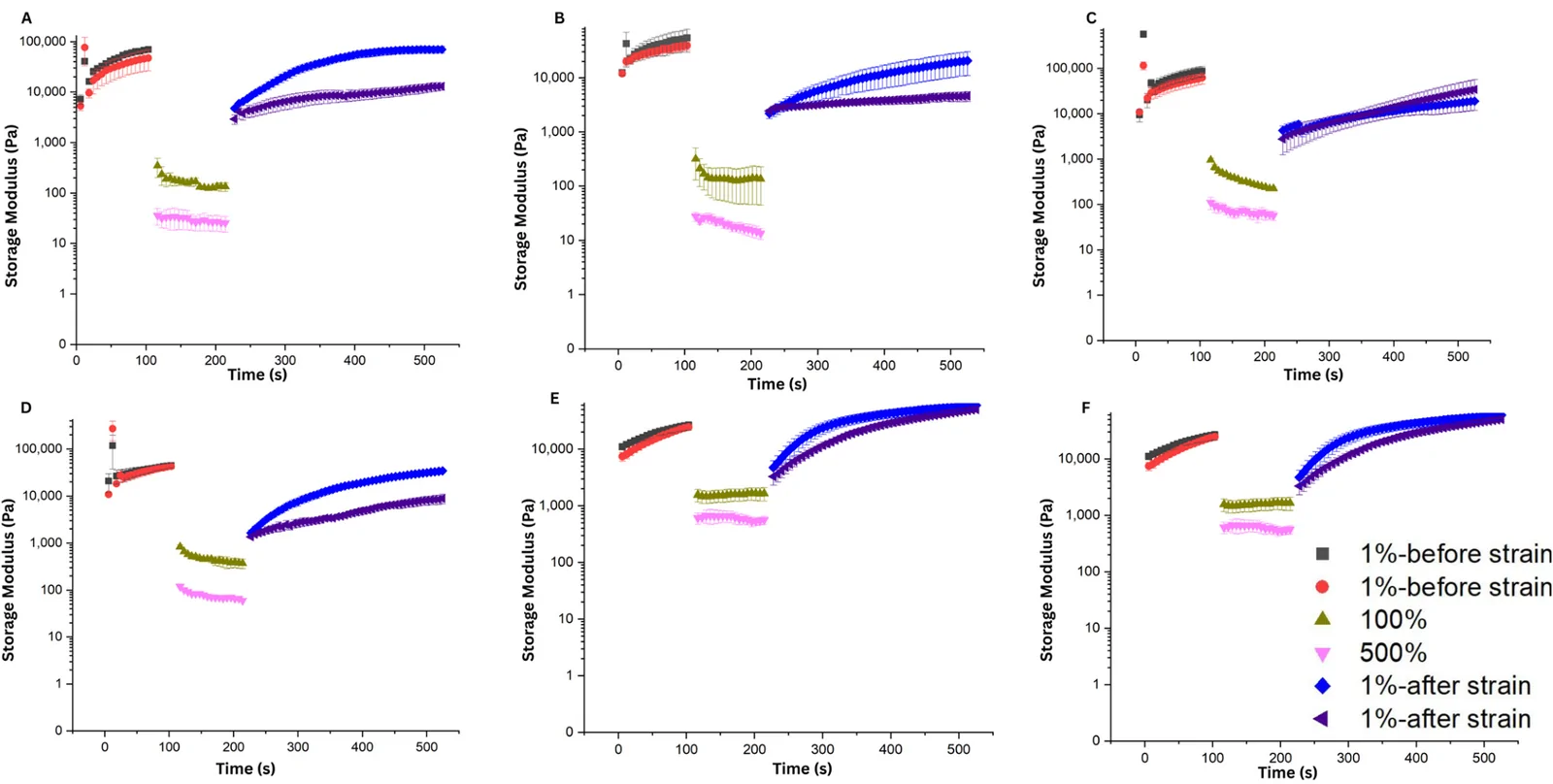
Recovery behavior of all scaffold compositions under cyclic strain. Storage modulus (G′) measured before high strain (1%), during high strain (100% and 500%), and after high strain (1%) for (**A**) P10B85F5, (**B**) P20B75F5, (**C**) P30B65F5, (**D**) P40B55F5, (**E**) P50B45F5 and (**F**) PCL. All compositions showed partial recovery of the viscoelastic structure following large-amplitude deformation. Data are presented as mean ± standard error.

Three-interval thixotropy tests (3ITT) were conducted to assess the structural breakdown and recovery capability of the PCL/β-TCP/Fe_3_O_4_ composite inks under high shear deformation, as shown in Figure 5. Across all formulations, there was a sharp drop in G′ during the high-strain phase (100% and 500%) due to structural breakdown, followed by partial restoration when the strain reverted to 1%. The time required to reach 80% of the original G′ value after shear served as a measure of recovery, and this property is crucial for applications in DIW. P30B65F5 exhibited recovery, reaching 80% of its original modulus in ~75 s under modest strain and ~130 s under high strain (500%). P20B75F5 recovered swiftly after lower strains but showed significant irreversibility after extensive shear, indicating that its network is easily disrupted beyond a critical threshold and cannot be reformed under mild post-shear conditions. This is evident as a lower final modulus after the recovery phase compared to its starting value. P40B55F5 exhibited slower recovery (taking 165–310 s to reach 80% recovery for 100% strain), indicating a denser structure. P10B85F5 and P50B45F5 showed fast recoveries for 100% strain but not for 500% strain.

#### 3.2.6. Gel Point

**Figure 6:**
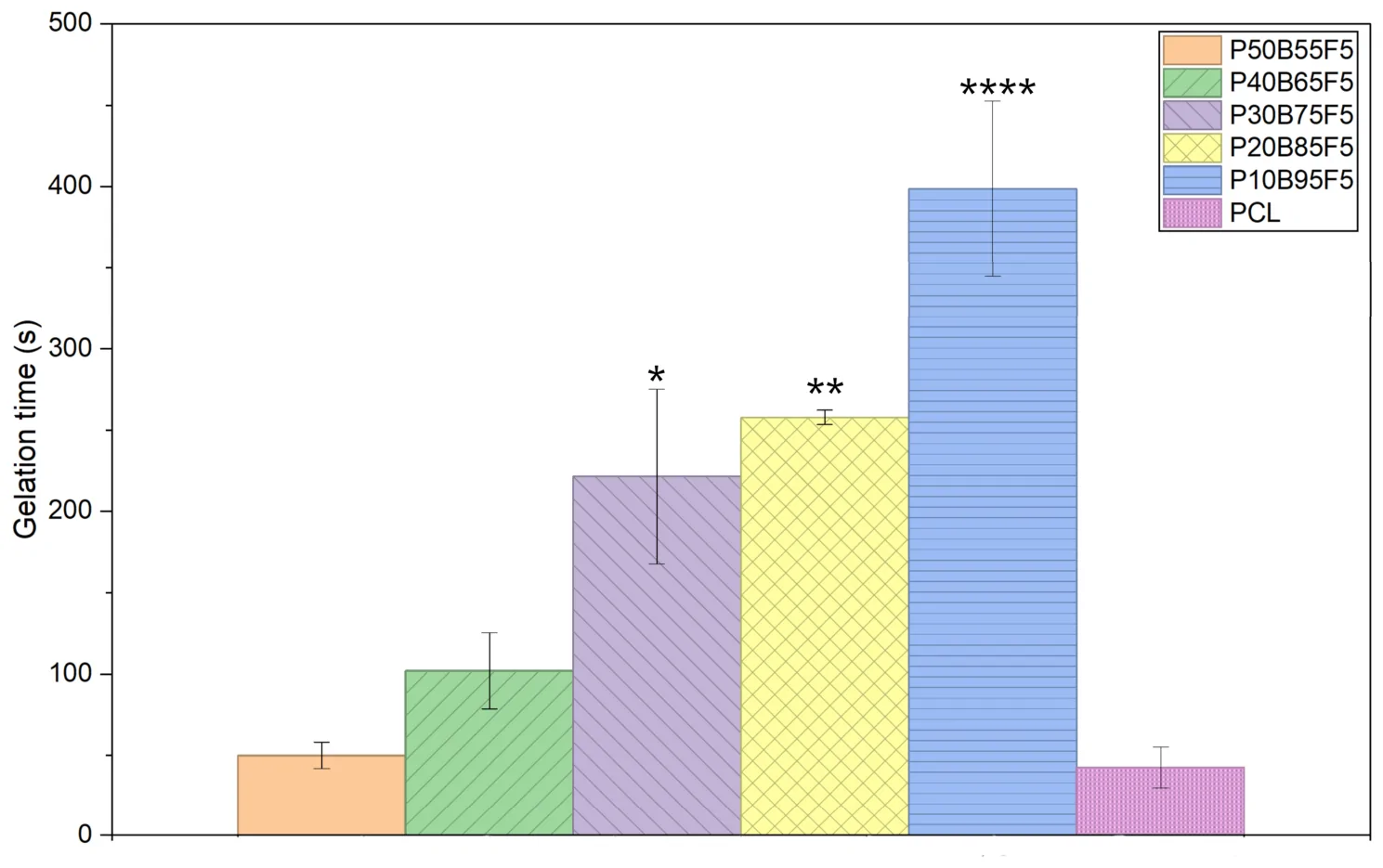
Gelation time of all formulated ink compositions, showing faster gelation with increasing ceramic content. Statistical significance was evaluated using one-way ANOVA followed by a multiple-comparisons test against the PCL control. Statistical significance relative to PCL is indicated as *p* < 0.05 (*), *p* < 0.01 (**), and *p* < 0.0001 (****). Data are presented as the mean ± standard error.

The gel point of a polymer is a critical stage in which the material transitions from a predominantly viscous (liquid-like) state to a predominantly elastic (solid-like) state. This transition is typically identified by the crossover of the storage modulus (G’) and loss modulus (G″) in rheological measurements. In the gelation tests (Figure 6), the time to solidification varied depending on the polymer composition, where P10B85F5 took the longest to solidify relative to PCL, whereas P50B45F5 and P50B45F5 gelation time was comparable to PCL.

### 3.3. 3D Printing of Formulated Inks

**Figure 7:**
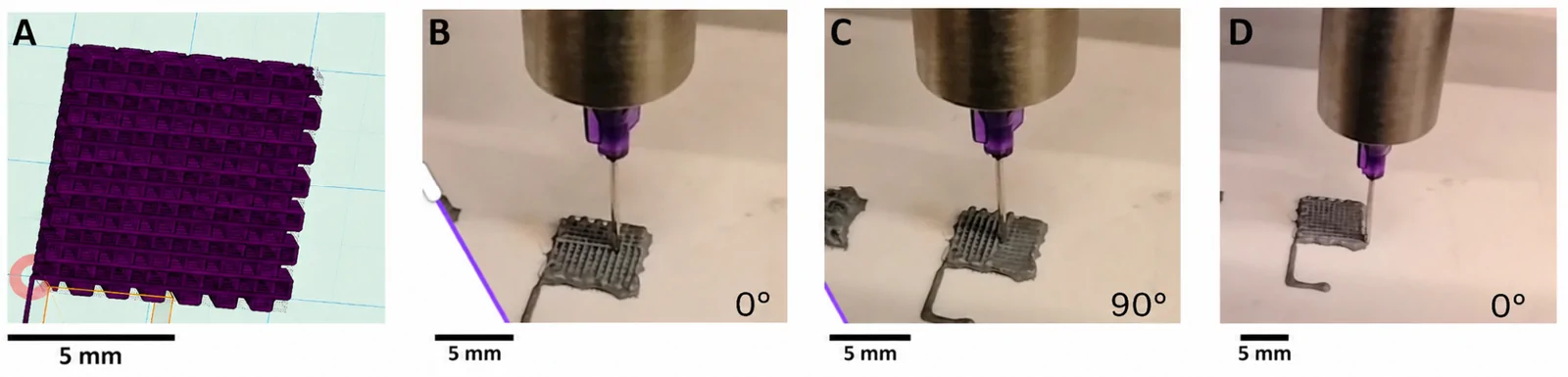
Fabrication of PCL/β-TCP/Fe_3_O_4_ composite scaffolds using DIW. (**A**) A CAD model illustrating the designed layer-by-layer lattice architecture. (**B**–**D**) Sequential DIW printing images of the P30B65F5 formulation using a 0.51 mm nozzle, demonstrating controlled filament deposition and 0–90–0° layer alignment. The temperature maintained while printing was 25 °C.

**Figure 8:**
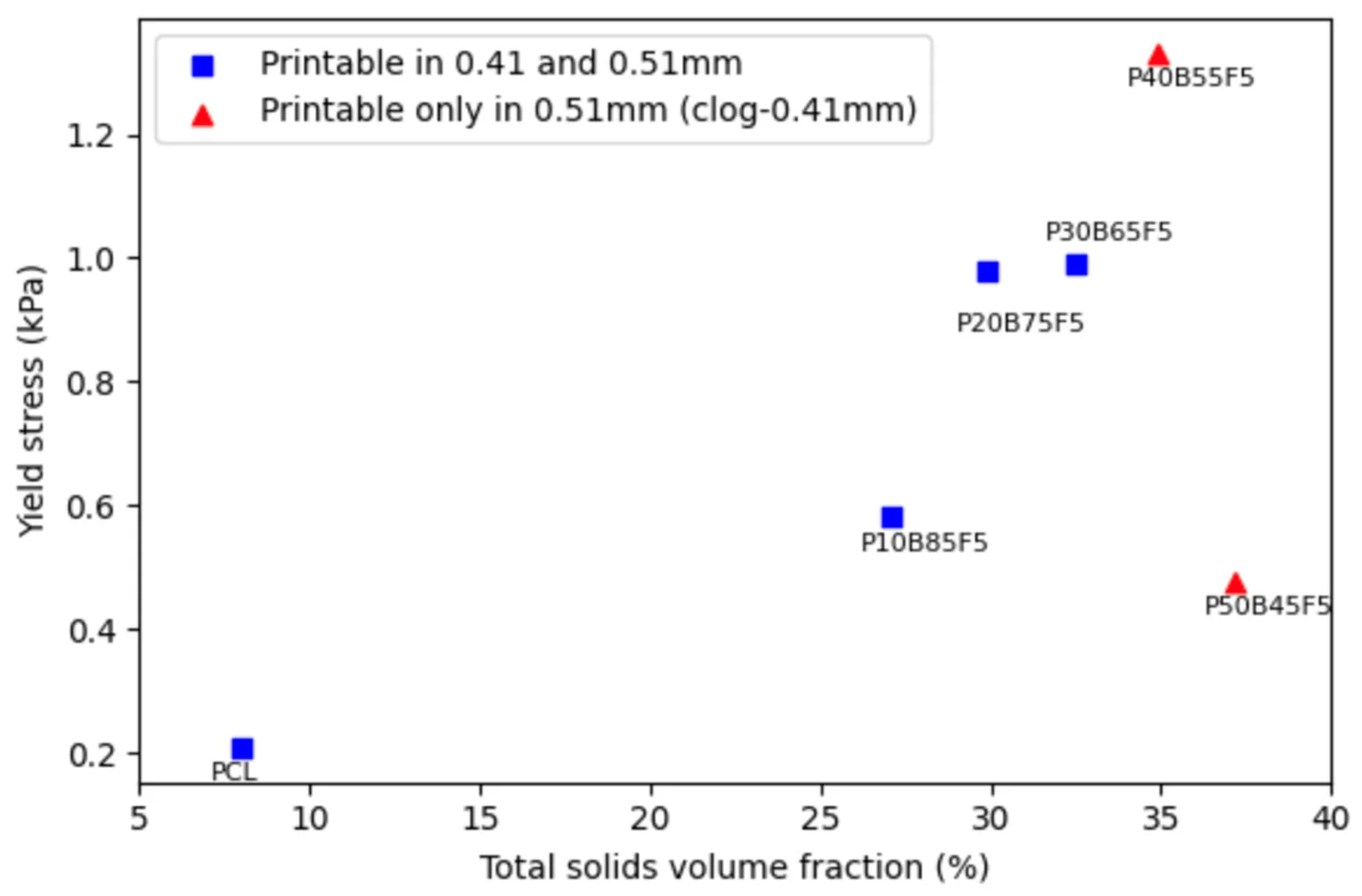
Nozzle compatibility map: blue squares denote inks that printed through both 0.41 mm and 0.51 mm nozzles, whereas red triangles represent inks that clogged in the 0.41 mm nozzle but remained printable in the 0.51 mm nozzle.

As indicated in Table 3, compositions ranging from P10B85F5 to P30B65F5 were successfully printed using the 0.41 mm diameter nozzle. However, as the polymer content increased, clogging was observed. On the other hand, for the 0.51 mm diameter nozzle, all the groups were printed and no clogging was observed. The samples displayed shape retention and layer adhesion, confirming that the DIW parameters and ink formulation enabled the reproducible fabrication of square-grid scaffolds (Figure 7) with varying polymer-to-ceramic ratios. Table 3 summarizes the weights of the scaffolds post-printing. Figure 8 summarizes the yield-stress vs. solids-fraction (%) map which shows that only intermediate compositions are printable in both nozzle diameters, confirming that nozzle compatibility is governed by the joint effect of yield stress and solid loading. In Figures S6 and S7, images of scaffolds fabricated by direct ink writing using 0.51 and 0.41 mm inner-diameter nozzles are presented.

### 3.4. SEM of the Printed Scaffolds

**Figure 9:**
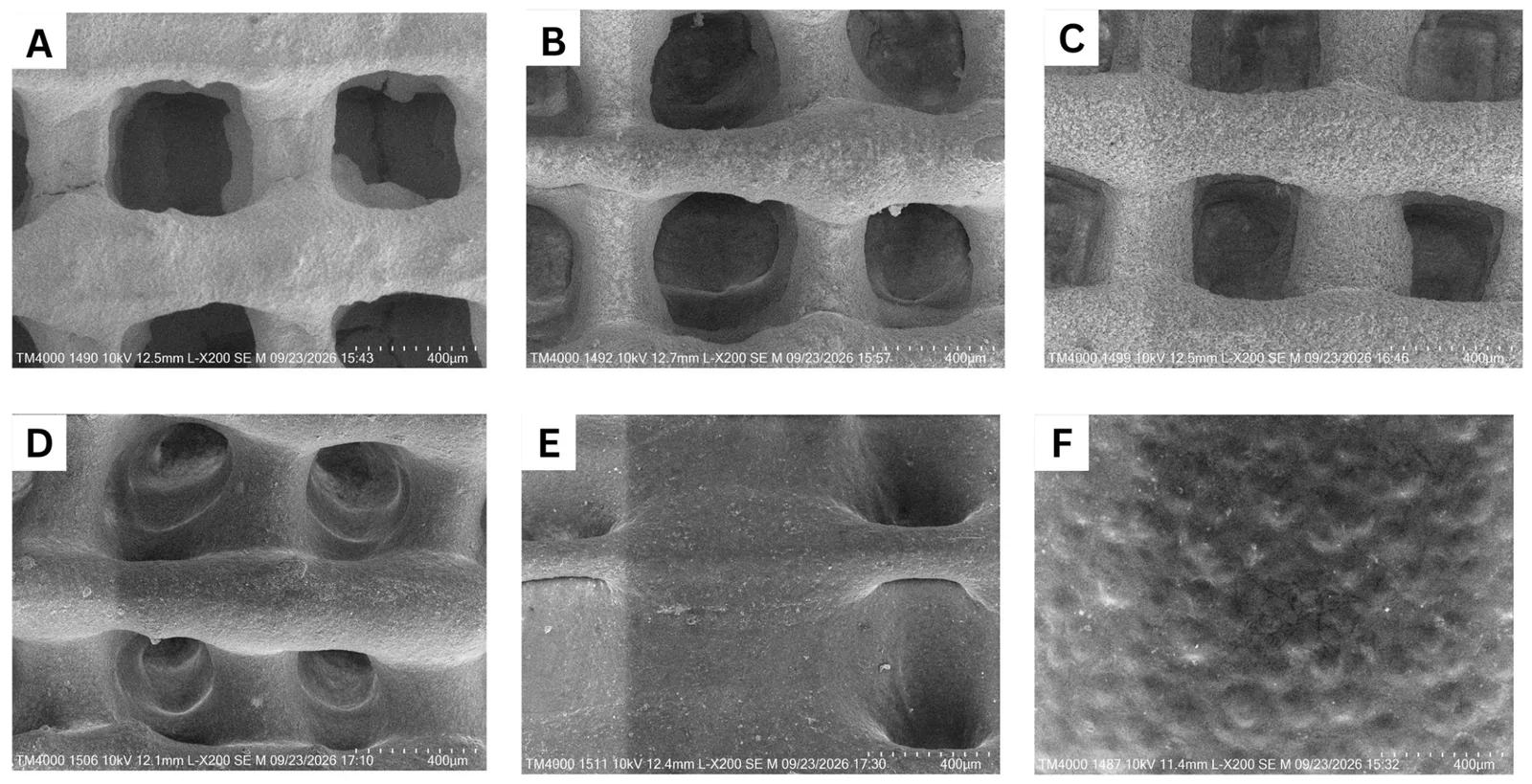
SEM of the surface morphology and pore/strand architecture of printed scaffolds. Representative micrographs of the top-most layer of (**A**) P10B85F5, (**B**) P20B75F5, (**C**) P30B65F5, (**D**) P40B55F5, (**E**) P50B45F5, and (**F**) PCL scaffolds. Higher-ceramic-content formulations exhibited more clearly defined printed strands and open-pore architecture, whereas increasing PCL content was associated with a progressive reduction in open pore definition and increased merging of adjacent strands. Images were acquired in secondary electron mode at 10 kV and 200× magnification. Scale bars = 400 µm.

SEM demonstrated composition-dependent differences in the surface morphology and pore/strand architecture of the printed scaffolds (Figure 9). The ceramic-rich formulations, particularly P10B85F5 and P20B75F5 (Figure 9A,B), exhibited clearly defined interconnected strands separated by large, open pores. The printed architecture remained readily distinguishable in P30B65F5 (Figure 9C), with comparatively well-defined strands and open pore spaces. As the PCL content increased, progressive changes in the printed architecture became apparent. P40B55F5 (Figure 9D) retained a recognizable porous structure; however, the pore openings appeared more rounded and less sharply defined than those observed in the lower-PCL formulations. In P50B45F5 (Figure 9E), the open-pore architecture was substantially reduced, with increased material agglomeration and merging between adjacent printed strands. The PCL scaffold (Figure 9F) exhibited the least distinct strand and pore architecture, with the top surface appearing comparatively continuous and lacking the clearly defined open pores observed in the ceramic-containing formulations. Overall, the SEM observations indicate that increasing PCL content was associated with a progressive reduction in the preservation of the designed open-pore architecture, whereas higher-ceramic-content formulations exhibited more clearly defined printed strands and pore boundaries.

### 3.5. XRD and FTIR of the Printed Scaffolds

**Figure 10:**
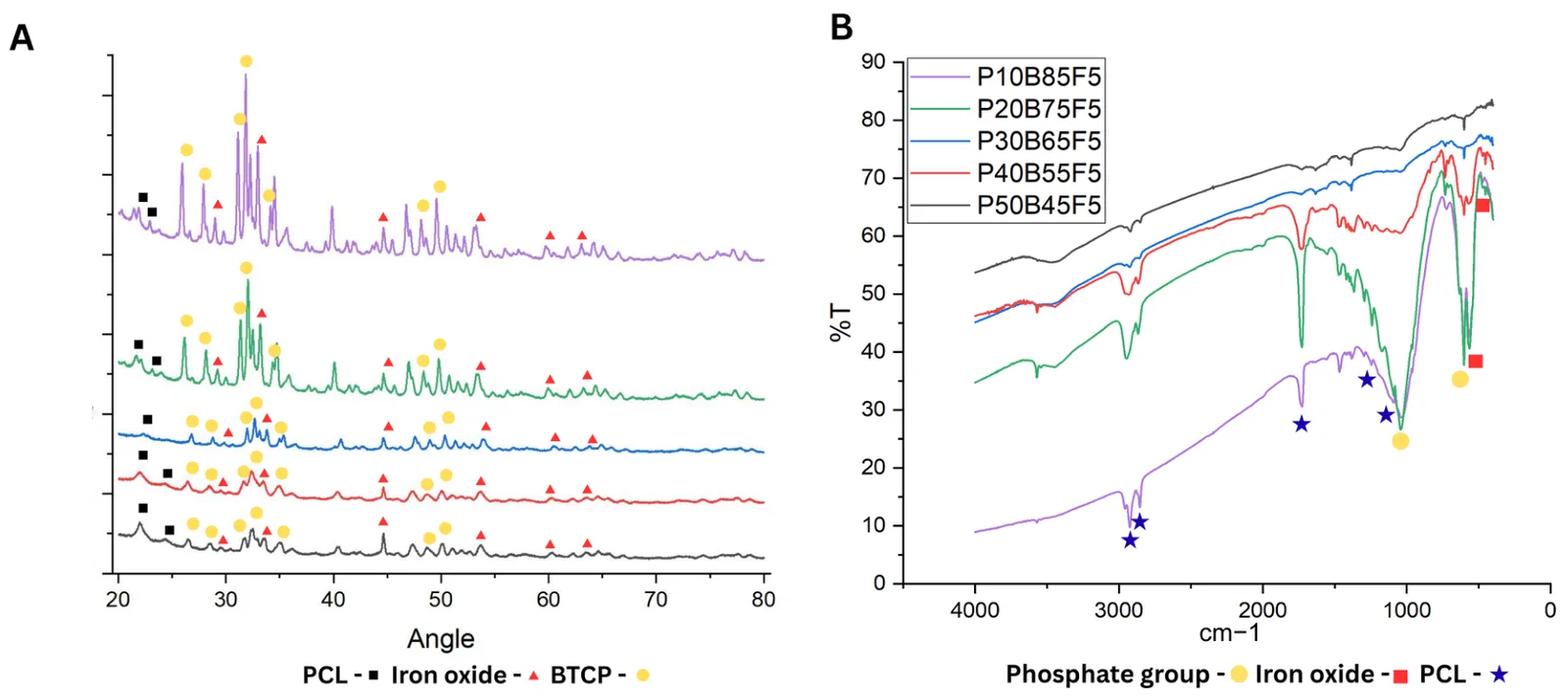
(**A**) XRD patterns of all scaffold compositions (P10B85F5, P20B75F5, P30B65F5, P40B55F5, P50B45F5). Characteristic peaks corresponding to PCL, β-TCP, and Fe_3_O_4_ are identified across samples. (**B**) FTIR spectra of scaffold compositions (P10B85F5, P20B75F5, P30B65F5, P40B55F5, P50B45F5), showing characteristic vibrational bands corresponding to PCL, β-TCP, and Fe_3_O_4_.

Representative XRD spectra of scaffold compositions (P10B85F5, P20B75F5, P30B65F5, P40B55F5, and P50B45F5) are shown in Figure 10A. Characteristic diffraction features corresponding to β-TCP, PCL, and Fe_3_O_4_ were identified in the composite scaffolds, indicating retention of the constituent phases following ink formulation and 3D printing. The XRD patterns were predominantly characterized by sharp and intense reflections associated with crystalline β-TCP. Characteristic β-TCP peaks were observed at approximately 26.0°, 27.8°, 31.0°, 32.4°, 34.4°, 48.4°, and 50.0°. In particular, the cluster of intense peaks within the ~31–34° region was prominent across the scaffold formulations and is characteristic of crystalline β-TCP [25,26]. In contrast, PCL exhibited broader and less intense diffraction features in the low 2θ region. The two principal PCL-associated peaks were identified at approximately 21.4° and 23.7°, corresponding to the (110) and (200) crystallographic planes, respectively [41].

Characteristic peaks associated with Fe_3_O_4_ were also present in the composite diffraction patterns. Magnetite exhibits characteristic peaks at 30.1°, 35.5°, 43.1°, 53.4°, 57.0°, and 62.6°, corresponding to the (220), (311), (400), (422), (511), and (440) crystallographic planes, respectively [42]. However, because Fe_3_O_4_ was maintained at 5 wt% in all compositions, several of its characteristic peaks occur close to the β-TCP peaks, and some Fe_3_O_4_-associated peaks were weak or overlapped with β-TCP. Differences in the relative peak intensities among the formulations were consistent with the systematic variations in the PCL/β-TCP ratio. The ceramic-rich compositions, particularly P10B85F5 and P20B75F5, exhibited more pronounced β-TCP-associated peaks, whereas increasing the PCL fraction produced a greater relative contribution from the broader polymer-associated features. These changes are consistent with the decrease in β-TCP content from 85 to 45 wt% and the corresponding increase in PCL content from 10 to 50 wt%, while the Fe_3_O_4_ concentration remained constant at 5 wt% [43,44].

FTIR spectra, shown in Figure 10B, displayed distinct absorption peaks that corresponded to characteristic vibrations of each component. For β- TCP, strong absorption bands appeared at 1050 cm^−1^ and 600 cm^−1^, reflecting phosphate group stretching and bending vibrations, respectively. The Fe_3_O_4_ phase showed peaks near 570 cm^−1^ and 450 cm^−1^, associated with Fe-O stretching vibration [45,46].

## 4. Discussion

The XRD and FTIR results confirm that the solvent-based gel-casting process preserved the crystalline β-TCP and Fe_3_O_4_ phases within the semi-crystalline PCL matrix, producing a multiphase polymer–ceramic–magnetic colloidal system suitable for rheological tuning and DIW processing [47,48,49]. GPC analysis showed a modest decrease in both the number-average and weight-average molecular weights of PCL after gel casting, indicating limited processing-induced chain scission and a broader molecular-weight distribution consistent with previous reports on biodegradable polyesters [50,51]. Within the framework of DIW, such modifications can affect chain entanglement density and segmental mobility, thereby impacting viscosity, yield stress, and viscoelastic recovery without compromising the overall integrity of the polymer matrix. In our system, the combination of preserved ceramic and magnetic crystallinity with a slightly broadened PCL molecular-weight distribution establishes the structural foundation for the composition-dependent rheology observed across the PCL/β-TCP/Fe_3_O_4_ series [52,53].

The behavior observed for all composite inks was a reduction in viscosity with increasing shear rate. It fulfills a key rheological requirement for DIW by enabling smooth extrusion through narrow nozzles. This response can be attributed to shear-induced molecular rearrangement of initially entangled polymer chains, which align along the flow direction under high shear, lowering intermolecular resistance and thus viscosity inside the nozzle [21]. Frequency sweep measurements indicated that both storage (G′) and loss (G″) moduli increased with oscillation frequency for all formulations, reflecting viscoelastic solids that stiffen under rapid deformation, consistent with the rheological profiles reported for PCL/calcium phosphate and hydrogel-based DIW systems. Notably, compositions such as P20B75F5 and P30B65F5 exhibited the highest G′ and G″ across the tested frequency range, indicative of a well-percolated filler–polymer network with efficient stress transfer, whereas P50B45F5 demonstrated markedly lower moduli, consistent with a polymer-dominated, more compliant structure [1,54,55]. The variation in tan δ with frequency underscores the composition-dependent viscoelastic balance of the inks, with extreme formulations (P10B85F5 and P50B45F5) showing viscous-dominated or brittle behavior at low frequency. However, intermediate compositions (P20B75F5, P30B65F5 and P40B55F5) approach tan δ ≈ 1 at higher frequencies, providing the balanced liquid-like flow in the nozzle and solid-like stability after deposition that is optimal for DIW.

These trends are consistent with previous research, which demonstrated that inks with medium tan δ values that become stiffer at higher frequencies are more capable of constructing and maintaining stable multilayer 3D-printed structures [53,54,56]. Amplitude sweep measurements revealed well-defined LVRs for all formulations, with stress-dependent decreases in G′ indicating network yielding, while the highest G′, broadest linear regimes, and peak yield stress for P30B65F5 and P40B55F5 point to better consistency in particle-filled DIW inks [57,58,59]. At low polymer contents, incomplete wetting of β-TCP and poor matrix continuity promote early network failure, whereas, at high polymer contents, the continuous PCL phase disrupts the load-bearing ceramic network, reducing frictional particle contacts and yield strength. The calculated yield pressures for the 0.51 mm nozzle (50–130 kPa) lie within typical DIW operating ranges, with a maximum value for P40B55F5 indicating strong particle jamming, and a sharp drop for P50B45F5 marking a transition to a more lubricated, polymer-dominated microstructure that extrudes easily but compromises shape fidelity. For the 0.41 mm nozzle, higher yield pressures (72–165 kPa) are required, yet formulations such as P10B85F5, P20B75F5, and P30B65F5 still fall within practical DIW operating window [60].

As the PCL content increases, the yield pressure also rises, eventually exceeding the DIW range of 40 to 140 kPa, as noted in previous studies [60]. For example, although the yield pressure for P50B45F5 remains within the range, clogging still occurs. Research on PCL-based and particle-filled inks indicates that increasing the filler size or volume fraction narrows the printable window, leading to filament discontinuities and nozzle clogging beyond certain thresholds [61,62,63]. This suggests that the clogging in polymer-rich compositions like P50B45F5 is less a result of rheological stiffness and more likely to be due to the high solid PCL particle fraction physically obstructing the nozzle, rather than the material’s resistance to flow initiation alone. This underscores that yield stress alone is insufficient as a predictor of printability, and that solid content and particle-size distribution must also be considered when selecting nozzle geometry for PCL-based composite inks. Transitioning from a 0.41 mm to a 0.51 mm nozzle, representing approximately a 24% increase in diameter, was sufficient to accommodate all formulations.

The recovery tests showed that all inks exhibit thixotropic, self-healing behavior, with G′ dropping under large strain and partially restoring when the strain is reduced, as expected for printable gels and pastes. Among them, P30B65F5 reached complete recovery in the least time after moderate and large strains (to about 80% of the initial G′), indicating a dynamic yet robust network well suited to repeated disruption and rebuilding during layer-by-layer deposition. In contract, P40B55F5 recovered more slowly relative to the extreme compositions (P10B85F5 and P50B45F5). These observations align with recent printability metrics that highlight the importance of optimizing both yield stress and recovery kinetics [64,65]. Gel-point analysis based on the G′–G″ crossover shows that higher-PCL formulations such as P50B45F5 reach the gel point more rapidly than low-PCL inks like P10B85F5, indicating faster evaporation-driven network locking in polymer-rich systems. In the context of nozzle-dependent printability, the yield-stress vs. solids-fraction (%) map shows that only intermediate ink formulations print reliably through both 0.41 and 0.51 mm nozzles. Whereas high-solids formulations (with either very high or very low yield stresses) clog in the smaller nozzle, indicating that nozzle compatibility arises from the combined effects of solids volume fraction and yield stress rather than either parameter alone [66,67]. In addition to the qualitative printing observations, the rheological data provide a basis for interpreting formulation-dependent extrusion resistance. In the present capillary-flow analysis, yield stress was used to estimate the pressure required to initiate flow through each nozzle geometry. However, this yield–pressure calculation does not represent the complete pressure required during steady DIW processing, because the latter may also depend on the high shear viscosity, printing flow rate, nozzle dimensions, and interfacial phenomena within the nozzle. Fatimi et al. similarly demonstrated, for injectable calcium phosphate suspensions, that rheological flow properties and capillary geometry can be used to predict injection pressure, while also showing that the agreement between predicted and measured pressures depends on the relationship between needle diameter and particle size [23]. In narrow flow channels, particle depletion and possible wall slip may cause experimental extrusion pressures to differ from theoretical predictions [23]. Accordingly, the calculated yield pressures reported here should be interpreted as first-order, comparative indicators of extrusion onset as opposed to direct measurements of the printing pressure required for continuous DIW deposition.

Fe_3_O_4_ was maintained at a constant concentration of 5 wt% in every formulation as the present experimental design was intended to isolate the influence of the PCL/β-TCP ratio on rheology and printability. Accordingly, the observed differences among formulations should be interpreted as composition-dependent effects of the polymer–ceramic balance at a fixed magnetic-particle loading. This design does not establish the independent contribution of Fe_3_O_4_ to viscosity, yield behavior, structural recovery, nozzle compatibility, or printing performance. Matched Fe_3_O_4_-free PCL/β-TCP controls will therefore be required in future work to determine the specific rheological, printing, magnetic, and biological effects of the magnetic phase.

This study has several limitations. First, we focused primarily on physicochemical and rheological characterization. Long-term mechanical behavior under physiological conditions, like heat and pressure, were not evaluated, and these are crucial for assessing the ultimate performance and biodegradability of scaffolds for biomedical applications. Future studies should therefore integrate dynamic mechanical analyses and degradation studies under simulated physiological environments. Additionally, other critical printing parameters, such as thermal changes in viscosity of the ink, were not considered in this study. While this allowed for the isolation of compositional effects, thermal control is known to significantly affect ink viscosity and print resolution, representing an important avenue for future optimization to expand the printability window and material versatility. Finally, the DIW experiments were limited to a single raster pattern (square-grid) architecture. However, quantitative printability assessments, including filament collapse, dimensional accuracy, pore fidelity, and systematic multilayer shape fidelity analyses, were not performed. While effective for validating fundamental rheological-printability correlations, the optimal printing parameters and material responses may vary significantly for more complex or intricate geometries, suggesting a need for further investigation across a broader range of scaffold designs. Overall, the findings identify intermediate PCL/β-TCP compositions, particularly P30B65F5 and P40B55F5, as promising candidates for further optimization because they achieved a favorable balance between extrusion behavior, viscoelastic response, and post-shear recovery. These results provide formulation guidelines for subsequent quantitative printability studies and biological evaluation of multifunctional DIW scaffolds.

## 5. Conclusions

This study establishes composition-dependent relationships among material formulation, rheological behavior, and preliminary DIW processability in multiphase PCL/β-TCP/Fe_3_O_4_ colloidal gels formulated for solvent-based extrusion. The successful extrusion and fabrication of square-grid structures from selected formulations provide preliminary evidence of DIW processability and the retention of the deposited architecture. Importantly, the results also indicate that extrusion behavior cannot be interpreted from a single rheological parameter such as yield stress alone. Rather, successful processing reflects the combined effects of yield behavior, viscoelasticity, structural recovery, composition, and nozzle geometry. These findings provide a rheological and processing framework for selecting polymer-to-ceramic ratios in highly filled PCL/β-TCP/Fe_3_O_4_ inks and identify intermediate compositions as the most promising candidates for further optimization.

The present findings should nevertheless be interpreted within the limitations of the study. The printing experiments provide evidence of preliminary DIW processability and nozzle compatibility but did not include quantitative filament collapse, dimensional accuracy, shape fidelity, or systematic multilayer analyses. Furthermore, biological performance was not evaluated; therefore, the investigated formulations should be considered as candidate multifunctional inks for the intended magnetic hyperthermia applications. Collectively, these findings provide composition-level design rules for tuning polymer-to-ceramic ratio and solid content to expand the printability window of PCL/β-TCP/Fe_3_O_4_ inks and lay the groundwork for future studies that couple these rheological metrics to quantitative mechanical performance and in vitro degradation under physiologic conditions.

## Figures and Tables

**Table 1:** Summary of the ink formulations used in this study.

Groups	Abbreviation
PCL(10%) + β-TCP (85%) + Fe_3_O_4_ (5%)	P10B85F5
PCL(20%) + β-TCP (75%) + Fe_3_O_4_ (5%)	P20B75F5
PCL(30%) + β-TCP (65%) + Fe_3_O_4_ (5%)	P30B65F5
PCL(40%) + β-TCP (55%) + Fe_3_O_4_ (5%)	P40B55F5
PCL(50%) + β-TCP (45%) + Fe_3_O_4_ (5%)	P50B45F5
PCL (10%)	PCL

**Table 2:** Rheological characterization of formulated ink compositions: yield stress, storage modulus, loss modulus, critical oscillation strain (%), and yield pressure values (for 0.51 mm and 0.41 mm internal tip diameters).

Group	Yield Stress (Pa)	Storage Modulus (Pa)	Loss Modulus (Pa)	Oscillation Strain (%)	ΔP_γ_ (kPa) (0.51 mm)	ΔP_γ_ (kPa) (0.41 mm)
P10B85F5	175.41 ± 26.13	2603.70 ± 353.36	5531.82 ± 1078.56	2.87 ± 0.07	17.47 ± 2.60	21.73 ± 3.24
P20B75F5	950.60 ± 81.67	18,349.40 ± 2500.26	27,916.93 ± 1976.88	2.85 ± 0.04	94.69 ± 8.13	117.78 ± 10.12
P30B65F5	1291.17 ± 350.63	8011.60 ± 2133.59	14,424.88 ± 3599.27	7.76 ± 0.32	128.61 ± 34.92	159.98 ± 43.45
P40B55F5	1331.10 ± 219.27	15,547.13 ± 3644.92	27,410.77 ± 4276.78	4.23 ± 0.04	132.58 ± 21.83	164.92 ± 27.17
P50B45F5	187.71 ± 69.35	3380.71 ± 1177.22	5338.47 ± 1761.21	2.79 ± 0.23	18.70 ± 6.91	23.26 ± 8.59
PCL	208.74 ± 177.75	1487.76 ± 177.28	3808.82 ± 1040.88	3.77 ± 2.81	20.79 ± 17.70	25.86 ± 22.02

**Table 3:** All group scaffolds average weight in grams.

Groups	P50B45F5	P40B55F5	P30B65F5	P20B75F5	P10B85F5
Wt. (0.41 mm)	NA	NA	188.4	197.4	288.96
Wt. (0.51 mm)	176.9	185.9	194.9	197.26	240.2

## Data Availability

The original contributions presented in this study are included in the article/Supplementary Materials. Further inquiries can be directed to the corresponding author.

## Supplementary Materials

The following supporting information can be downloaded at: https://www.mdpi.com/article/10.3390/ma19214496/s1. Figure S1: Schematic of iron oxide particle synthesis. FeCl_3_·6H_2_O, FeCl_2_·4H_2_O, HCl, and water were mixed to form the precursor, which was subsequently subjected to NaOH precipitation, centrifugation, vacuum drying at 60 °C, and sieving to obtain iron oxide particles with a diameter <37 µm; Figure S2: Schematic overview of the material preparation workflows; Figure S3: SEM analysis of β-TCP powder yielded a pooled number-based ECD distribution comprising 5781 segmented particulate features from five analyzed fields. The mean ECD was 6.12 µm, with a median (P50) of 5.46 µm. The distribution was right-skewed, with the majority of measured features concentrated below approximately 10 µm and a smaller population extending toward larger ECD values; Figure S4: Storage modulus (G′) of all formulated ink compositions as a function of oscillation stress, showing the stress-dependent viscoelastic response and the onset of structural softening across compositions, with data being presented as mean ± standard error; Figure S5: Amplitude sweep and yield behavior of composite scaffold inks. Panels A1–F1 show the amplitude sweep results identifying the linear viscoelastic region (LVR), G’, loss modulus (G”), and the onset of nonlinearity, with yield points marked by dashed vertical lines. Panels A2–F2 present the corresponding oscillatory (true) stress as a function of oscillatory strain, with yield stress indicated for each formulation. A-P10B85F5, B-P20B75F5, C-P30B65F5, D-P40B55F5, E-P50B45F5 and F-PCL. Sample size n = 3 and mean; Figure S6: Representative images of scaffolds fabricated by direct ink writing using a 0.51 mm inner-diameter nozzle: (A) PCL, (B) P10B85F5, (C) P20B75F5, (D) P30B65F5, (E) P40B55F5, and (F) P50B45F5; Figure S7: Representative images of scaffolds fabricated by direct ink writing using a 0.41 mm inner-diameter nozzle: (A) P10B85F5, (B) P20B75F5, and (C) P30B65F5; Table S1: One-way ANOVA of rheological parameters for PCL and PCL/β-TCP/Fe_3_O_4_ formulations (n = 3 per group); Table S2: Tukey HSD pairwise comparisons. Statistical significance is indicated as *p* < 0.05 (*), *p* < 0.01 (**), *p* < 0.001 (***), and *p* < 0.0001 (****); ns indicates *p* ≥ 0.05. Script S1: Particle analysis.

## References

[1] Rau D.A., Bortner M.J., Williams C.B. (2023). A rheology roadmap for evaluating the printability of material extrusion inks. Addit. Manuf..

[2] Saadi M.A.S.R., Maguire A., Pottackal N.T., Thakur S.H., Ikram M.M., Hart A.J., Ajayan P.M., Rahman M.M. (2022). Direct Ink Writing: A 3D Printing Technology for Diverse Materials. Adv. Mater..

[3] M’bArki A., Bocquet L., Stevenson A. (2017). Linking Rheology and Printability for Dense and Strong Ceramics by Direct Ink Writing. Sci. Rep..

[4] Karyappa R., Hashimoto M. (2019). Chocolate-based Ink Three-dimensional Printing (Ci3DP). Sci. Rep..

[5] Li C., Feng C., Zhang L., Zhang L., Wang L. (2025). Direct ink writing of polymer-based materials—A review. Polym. Eng. Sci..

[6] Ni X., Feng J., Liang M., Zhou F., Xia Y., Dong Z., Xue Q., Li Z., Pu F., Xia P. (2025). Enhancing Bone Repair with β-TCP-Based Composite Scaffolds: A Review of Design Strategies and Biological Mechanisms. Orthop. Res. Rev..

[7] Hesaraki S., Saba G., Shahrezaee M., Nezafati N., Orshesh Z., Roshanfar F., Borhan S., Glasmacher B., Makvandi P., Xu Y. (2024). Reinforcing β-tricalcium phosphate scaffolds for potential applications in bone tissue engineering: Impact of functionalized multi-walled carbon nanotubes. Sci. Rep..

[8] Lu H., Zhou Y., Ma Y., Xiao L., Ji W., Zhang Y., Wang X. (2021). Current Application of Beta-Tricalcium Phosphate in Bone Repair and Its Mechanism to Regulate Osteogenesis. Front. Mater..

[9] Fu Q., Saiz E., Tomsia A.P. (2011). Direct Ink Writing of Highly Porous and Strong Glass Scaffolds for Load-bearing Bone Defects Repair and Regeneration. Acta Biomater..

[10] Chen Z., He P., Ouyang J., Duan X., Jia D., Colombo P., Zhou Y. (2026). Direct ink writing of ceramics: From fundamentals to industrial applications. Adv. Mater. Join..

[11] del-Mazo-Barbara L., Ginebra M.-P. (2021). Rheological characterisation of ceramic inks for 3D direct ink writing: A review. J. Eur. Ceram. Soc..

[12] Nayak V.V., Sanjairaj V., Behera R.K., Smay J.E., Gupta N., Coelho P.G., Witek L. (2024). Direct inkjet writing of polylactic acid/β-tricalcium phosphate composites for bone tissue regeneration: A proof-of-concept study. J. Biomed. Mater. Res. Part B Appl. Biomater..

[13] Gharibshahian M., Salehi M., Beheshtizadeh N., Kamalabadi-Farahani M., Atashi A., Nourbakhsh M.-S., Alizadeh M. (2023). Recent advances on 3D-printed PCL-based composite scaffolds for bone tissue engineering. Front. Bioeng. Biotechnol..

[14] Zhang C., Qi Y., Dai B., Lin R., You J., Wang Y., Xing S., Ye Y., Li D.-J. (2026). Preparation and characterization of PCL@β-TCP composite antibacterial scaffolds by additive manufacturing. J. Appl. Biomater. Funct. Mater..

[15] Rivera D., Bouras A., Mattioli M., Anastasiadou M., Pacentra A.C., Pelcher O., Koziel C., Schupper A.J., Chanenchuk T., Carlton H. (2025). Magnetic hyperthermia therapy enhances the chemoradiosensitivity of glioblastoma. Sci. Rep..

[16] Zhang B., Chung S.H., Barker S., Craig D., Narayan R.J., Huang J. (2021). Direct ink writing of polycaprolactone/polyethylene oxide based 3D constructs. Prog. Nat. Sci. Mater. Int..

[17] Miranda P., Saiz E., Gryn K., Tomsia A.P. (2005). Sintering and robocasting of β-tricalcium phosphate scaffolds for orthopaedic applications. Acta Biomater..

[18] Zhang Y., Zhai D., Xu M., Yao Q., Chang J., Wu C. (2016). 3D-printed bioceramic scaffolds with a Fe_3_O_4_/graphene oxide nanocomposite interface for hyperthermia therapy of bone tumor cells. J. Mater. Chem. B.

[19] Zhang J., Zhao S., Zhu M., Zhu Y., Zhang Y., Liu Z., Zhang C. (2014). 3D-printed magnetic Fe_3_O_4_/MBG/PCL composite scaffolds with multifunctionality of bone regeneration, local anticancer drug delivery and hyperthermia. J. Mater. Chem. B.

[20] Jeon M., Halbert M.V., Stephen Z.R., Zhang M. (2021). Iron Oxide Nanoparticles as T 1 Contrast Agents for Magnetic Resonance Imaging: Fundamentals, Challenges, Applications, and Prospectives. Adv. Mater..

[21] Wu K., Liu J., Saha R., Peng C., Su D., Wang Y.A., Wang J.-P. (2021). Investigation of Commercial Iron Oxide Nanoparticles: Structural and Magnetic Property Characterization. ACS Omega.

[22] Malhotra N., Lee J.-S., Liman R.A.D., Ruallo J.M.S., Villaflores O.B., Ger T.-R., Hsiao C.-D. (2020). Potential Toxicity of Iron Oxide Magnetic Nanoparticles: A Review. Molecules.

[23] Fatimi A., Tassin J.-F., Bosco J., Deterre R., Axelos M.A.V., Weiss P. (2012). Injection of calcium phosphate pastes: Prediction of injection force and comparison with experiments. J. Mater. Sci. Mater. Med..

[24] Figueiredo V.V., Bonfim R.P.F., Silva M.A., Moreira C.R., Pérez G.A.C., Lourenço W.A., Salles B.R., Salim V.M.M. (2025). Iron oxide nanoparticles: Synthesis, surface reactivity and transformative behavior under methane reducing atmosphere study via in situ XRD. Mater. Chem. Phys..

[25] Ruiz-Aguilar C., Olivares-Pinto U., Aguilar-Reyes E.A., López-Juárez R., Alfonso I. (2018). Characterization of β-tricalcium phosphate powders synthesized by sol–gel and mechanosynthesis. Bol. Soc. Esp. Ceram. Vidr..

[26] Nahar U.K., Shovon B., Chandra R.D., Chandra S.P., Shukanta B., Muhammed Y.M., Sydul Islam M.D. (2017). Characterization of Beta-Tricalcium Phosphate (β- TCP) Produced at Different Process Conditions. J. Bioeng. Biomed. Sci..

[27] Rehman W., Masood S., Begum S., Chandio A.D., Khan S.R., Gulnar L., Khattak R., Khan M.S. (2021). Viscometric and FTIR studies of chloroquine phosphate, acefylline piperazine and gentamicin sulfate in aqueous-polyethylene glycol and aqueous-polyvinyl pyrrolidone at different temperatures. Arab. J. Chem..

[28] Petráková M., Gorejová R., Shepa J., Macko J., Kupková M., Petruš O., Baláž M., Sopčák T., Mičušík M., Kožár M. (2024). Effect of Gentamicin-Loaded Calcium Phosphate Coating and Polymeric Coating on the Degradation Properties of Biodegradable Iron-Based Biomaterials. ACS Omega.

[29] Shingaki S., Takada M., Sasai K., Bibi R., Kobayashi T., Nomura T., Saito C. (2003). Impact of lymph node metastasis on the pattern of failure and survival in oral carcinomas. Am. J. Surg..

[30] ShiraliPour F., Shafiei S.S., Nikakhtar Y. (2021). Three-dimensional porous poly(ε-caprolactone)/beta-tricalcium phosphate microsphere-aggregated scaffold for bone tissue engineering. Int. J. Appl. Ceram. Technol..

[31] Park S.A., Lee H.-J., Kim S.-Y., Kim K.-S., Jo D.-W., Park S.-Y. (2021). Three-dimensionally printed polycaprolactone/beta-tricalcium phosphate scaffold was more effective as an rhBMP -2 carrier for new bone formation than polycaprolactone alone. J. Biomed. Mater. Res. Part A.

[32] Kim S.I., Kim N.E., Park S., Choi J.H., Lee Y., Jeon G., Song J.E., Khang G. (2022). Characterization of non-solvent- and thermal-induced phase separation applied polycaprolactone/demineralized bone matrix scaffold for bone tissue engineering. In Vitr. Model.

[33] Guan S., Liu X., Zhang Y., Liu Y., Wang L., Liu Y. (2019). Synthesis and Characterization of Polycaprolactone Modified Trimellitate Nano-Lubricant. Materials.

[34] Namikuchi E.A., Gaspar R.D.L., da Silva D.S., Raimundo I.M., OMazali I. (2021). PEG size effect and its interaction with Fe_3_O_4_ nanoparticles synthesized by solvothermal method: Morphology and effect of pH on the stability. Nano Express.

[35] Hussain A., Yasar M., Ahmad G., Ijaz M., Aziz A., Nawaz M.G., Khan F.A., Iqbal H., Shakeel W., Momand H. (2023). Synthesis, characterization, and applications of iron oxide nanoparticles. Int. J. Health Sci..

[36] Ahmed M., Zayed M., El-Dek S., Hady M.A., El Sherbiny D.H., Uskoković V. (2021). Nanofibrous ε-polycaprolactone scaffolds containing Ag-doped magnetite nanoparticles: Physicochemical characterization and biological testing for wound dressing applications in vitro and in vivo. Bioact. Mater..

[37] Deschamps I.S., Magrin G.L., Magini R.S., Fredel M.C., Benfatti C.A., Souza J.C. (2017). On the synthesis and characterization of β-tricalcium phosphate scaffolds coated with collagen or poly (D, L-lactic acid) for alveolar bone augmentation. Eur. J. Dent..

[38] Ahghari M.R., Amiri-Khamakani Z., Maleki A. (2023). Synthesis and characterization of Se doped Fe_3_O_4_ nanoparticles for catalytic and biological properties. Sci. Rep..

[39] Liu Z.-H., Li Y., Zhang C.-J., Zhang Y.-Y., Cao X.-H., Zhang X.-H. (2020). Synthesis of high-molecular-weight poly(ε-caprolactone) via heterogeneous zinc-cobalt(III) double metal cyanide complex. Giant.

[40] Paul D L B., Praveen A.S., Čepová L., Elangovan M. (2023). Rheological Behavior and Printability Study of Tri-Calcium Phosphate Ceramic Inks for Direct Ink Writing Method. Polymers.

[41] An C., Oyon S.A., Zhang F., Na H., Carrier J., Radu D., Lai C.-Y. (2025). Bioactive Electrospun Polycaprolactone Films with Gallic Acid-Loaded Mesoporous Silica Nanoplatelets for Enhanced Seed Germination. ACS Mater. Au.

[42] Soukup D., Moise S., Céspedes E., Dobson J., Telling N.D. (2015). In Situ Measurement of Magnetization Relaxation of Internalized Nanoparticles in Live Cells. ACS Nano.

[43] Karimzadeh I., Aghazadeh M., Doroudi T., Ganjali M.R., Kolivand P.H. (2017). Superparamagnetic Iron Oxide (Fe_3_O_4_) Nanoparticles Coated with PEG/PEI for Biomedical Applications: A Facile and Scalable Preparation Route Based on the Cathodic Electrochemical Deposition Method. Adv. Phys. Chem..

[44] Tavares D.d.S., Castro L.d.O., Soares G.D.d.A., Alves G.G., Granjeiro J.M. (2013). Synthesis and cytotoxicity evaluation of granular magnesium substituted β-tricalcium phosphate. J. Appl. Oral Sci..

[45] Ngo S.T., Lee W.-F., Wu Y.-F., Salamanca E., Aung L.M., Chao Y.-Q., Tsao T.-C., Hseuh H.-W., Lee Y.-H., Wang C.-C. (2023). Fabrication of Solvent-Free PCL/β-TCP Composite Fiber for 3D Printing: Physiochemical and Biological Investigation. Polymers.

[46] Aydogdu M.O., Mutlu B., Kurt M., Inan A.T., Kuruca S.E., Erdemir G., Sahin Y.M., Ekren N., Oktar F.N., Gunduz O. (2019). Developments of 3D polycaprolactone/beta-tricalcium phosphate/collagen scaffolds for hard tissue engineering. J. Aust. Ceram. Soc..

[47] Zheng C., Zhang M. (2023). 3D-printed PCL/β-TCP/CS composite artificial bone and histocompatibility study. J. Orthop. Surg. Res..

[48] Wang Q., Ye W., Ma Z., Xie W., Zhong L., Wang Y., Rong Q. (2021). 3D printed PCL/β-TCP cross-scale scaffold with high-precision fiber for providing cell growth and forming bones in the pores. Mater. Sci. Eng. C.

[49] Jang J.-W., Min K.-E., Kim C., Wern C., Yi S. (2024). Rheological Properties and 3D Printing Behavior of PCL and DMSO_2_ Composites for Bio-Scaffold. Materials.

[50] Velghe I., Buffel B., Vandeginste V., Thielemans W., Desplentere F. (2023). Review on the Degradation of Poly(lactic acid) during Melt Processing. Polymers.

[51] Marsh D.S. (2020). Understanding and Minimizing Polycaprolactone Degradation During Processing Using Rheology and Multi-Detector GPC. https://analyzing-testing.netzsch.com/en-US/application-literature/understanding-and-minimizing-polycaprolactone-degradation-during-processing-using-rheology-and-multi-detector-gpc.

[52] Salehabadi M., Mirzadeh H. (2025). 3D Printing of Polyester Scaffolds for Bone Tissue Engineering: Advancements and Challenges. Adv. Mater. Technol..

[53] Del-Mazo-Barbara L., Ginebra M.-P. (2024). Self-hardening polycaprolactone/calcium phosphate inks for 3D printing of bone scaffolds: Rheology, mechanical properties and shelf-life. Mater. Des..

[54] Wei P., Cipriani C., Hsieh C.-M., Kamani K., Rogers S., Pentzer E. (2023). Go with the flow: Rheological requirements for direct ink write printability. J. Appl. Phys..

[55] Calafel M.I., Criado-Gonzalez M., Aguirresarobe R., Fernández M., Mijangos C. (2025). From rheological concepts to additive manufacturing assessment of hydrogel-based materials for advanced bioprinting applications. Mater. Adv..

[56] Kumar P., Ray R., Kumar S., Powar S., Doddamani M. (2026). A review on direct ink writing 3D printing: Materials, properties, and future advancements. Prog. Eng. Sci..

[57] Gillispie G.J., Copus J., Uzun-Per M., Yoo J.J., Atala A., Niazi M.K.K., Lee S.J. (2023). The correlation between rheological properties and extrusion-based printability in bioink artifact quantification. Mater. Des..

[58] Germanton G., Martin K.L., Hossain A., Posey N.D., Ponder J.F., Ramirez C., Gnanasekar P., Wiegart L., Polisetty P., Hallinan D.T. (2024). Insights into Preceramic Polymer-Based Additive Manufacturing Inks via Rheological and Scattering Studies of Preceramic Polymer-Grafted Nanoparticles Suspended in Polycarbosilane. ACS Appl. Eng. Mater..

[59] Legett S.A., Torres X., Schmalzer A.M., Pacheco A., Stockdale J.R., Talley S., Robison T., Labouriau A. (2022). Balancing Functionality and Printability: High-Loading Polymer Resins for Direct Ink Writing. Polymers.

[60] Arjoca S., Bojin F., Neagu M., Păunescu A., Neagu A., Păunescu V. (2024). Hydrogel Extrusion Speed Measurements for the Optimization of Bioprinting Parameters. Gels.

[61] Zhang W., Ullah I., Shi L., Zhang Y., Ou H., Zhou J., Ullah M.W., Zhang X., Li W. (2019). Fabrication and characterization of porous polycaprolactone scaffold via extrusion-based cryogenic 3D printing for tissue engineering. Mater. Des..

[62] Yan M., Awad H.A. (2023). Optimizing rheological properties for printability: Low-temperature extrusion 3D printing of hydroxyapatite-polycaprolactone mixture inks for bone tissue engineering. Front. Mater..

[63] Beran T., Mulholland T., Henning F., Rudolph N., Osswald T.A. (2018). Nozzle clogging factors during fused filament fabrication of spherical particle filled polymers. Addit. Manuf..

[64] Cooke M.E., Rosenzweig D.H. (2021). The rheology of direct and suspended extrusion bioprinting. APL Bioeng..

[65] Özdemir Z., Šaman D., Bertula K., Lahtinen M., Bednárová L., Pazderková M., Rárová L., Nonappa, Wimmer Z. (2021). Rapid Self-Healing and Thixotropic Organogelation of Amphiphilic Oleanolic Acid–Spermine Conjugates. Langmuir.

[66] Herrada-Manchón H., Fernández M.A., Aguilar E. (2023). Essential Guide to Hydrogel Rheology in Extrusion 3D Printing: How to Measure It and Why It Matters?. Gels.

[67] Rau D.A., Williams C.B., Bortner M.J. (2023). Rheology and printability: A survey of critical relationships for direct ink write materials design. Prog. Mater. Sci..

