# Supplementary material for: Physicochemical and Rheological Characterization of Ferrimagnetic Polymer–Ceramic Colloidal Gels as Potential Inks for Direct Ink Writing
Source: Materials (Basel). 2026 Oct 26;19(21):4496. doi: 10.3390/ma19214496 (PMC13763731; doi:10.3390/ma19214496)
Supplement: Supplementary file 1 [file materials-19-04496-s001.zip › materials-4545283-supplementary.pdf]

Supplementary Figures and Tables

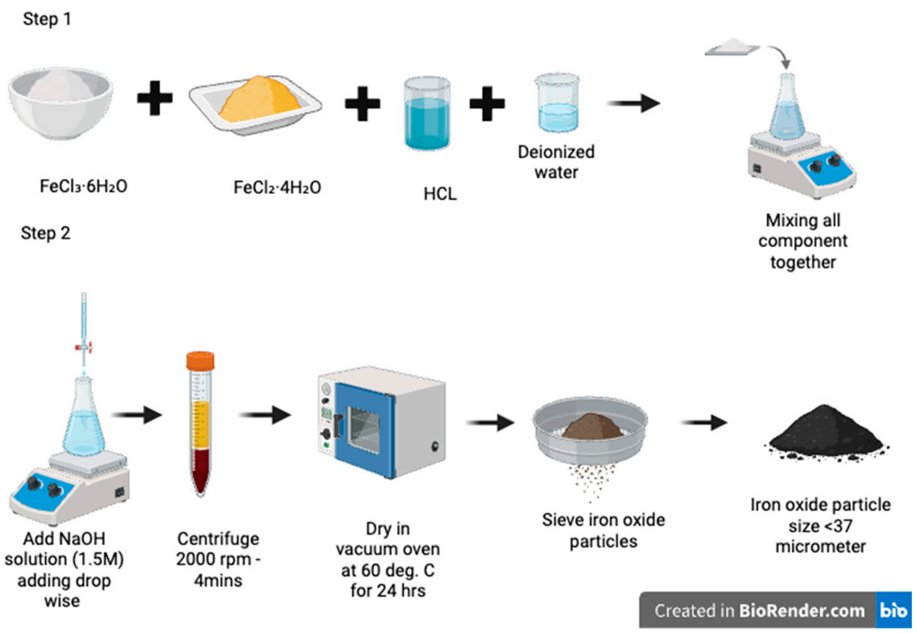

Figure S1: Schematic of iron oxide particle synthesis. $FeCl_3{\cdot}6H_2O$, $FeCl_2{\cdot}4H_2O$, HCl, and water were mixed to form the precursor, which was subsequently subjected to NaOH precipitation, centrifugation, vacuum drying at 60°C, and sieving to obtain iron oxide particles with a diameter <37 µm. (Created by BioRender)

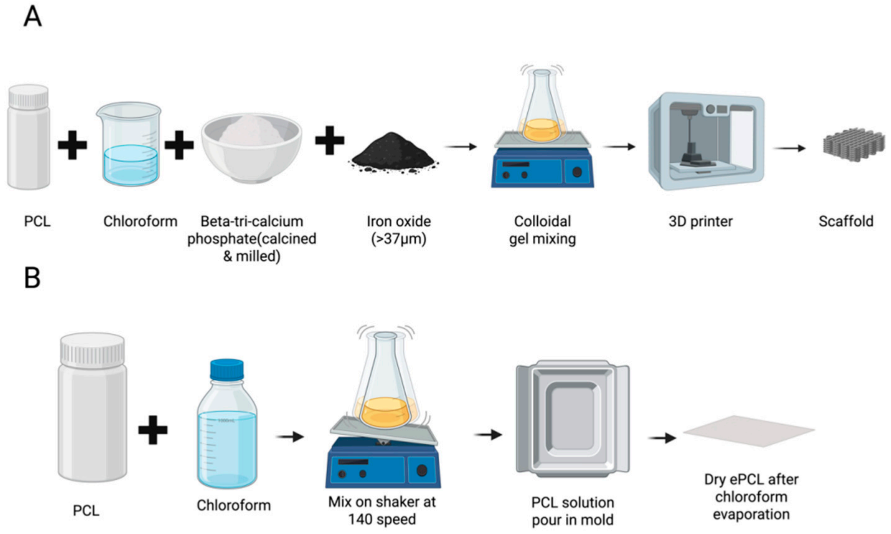

Figure S2: Schematic illustration of the preparation of polymer–ceramic colloidal gels and evaporated polycaprolactone (ePCL) samples. (A) Preparation of magnetic polymer–ceramic colloidal gels by combining polycaprolactone (PCL), chloroform, calcined and milled β-tricalcium phosphate (β-TCP), and iron oxide (Fe_3O_4) particles, followed by mixing to obtain a homogeneous colloidal gel suitable for direct ink writing (DIW) and subsequent fabrication of three-dimensional scaffolds. (B) Preparation of evaporated PCL (ePCL) samples by dissolving PCL in chloroform under continuous shaking at 140 rpm, followed by casting the polymer solution into a mold and allowing the chloroform to evaporate, resulting in solid ePCL specimens. (Created by BioRender)

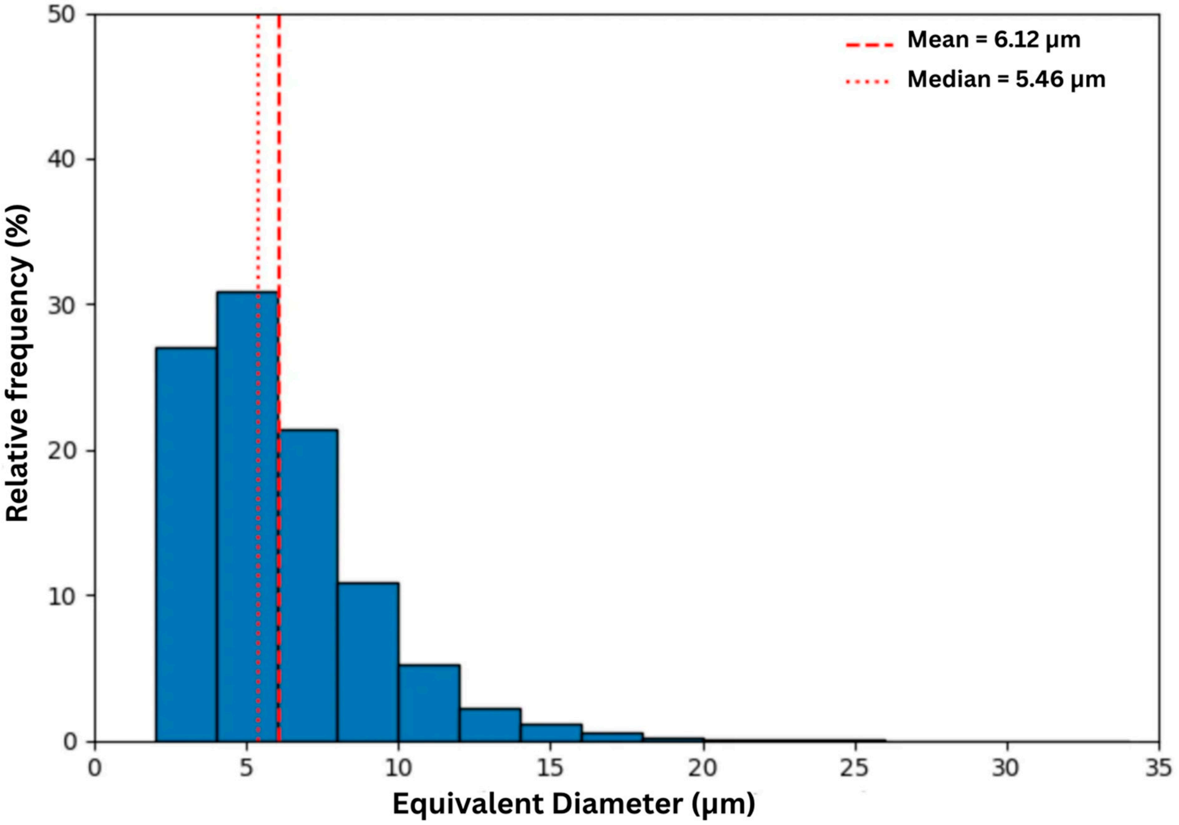

Figure S3: SEM analysis of β-TCP powder yielded a pooled number-based ECD distribution comprising 5,781 segmented particulate features from five analyzed fields. The mean ECD was 6.12 µm, with a median (P50) of 5.46 µm. The particle-size distribution was right-skewed, with approximately 90% of the segmented features exhibiting equivalent circular diameters below 10 µm, while the remaining ~10% formed larger diameters, the largest diameter being 32.53 µm.

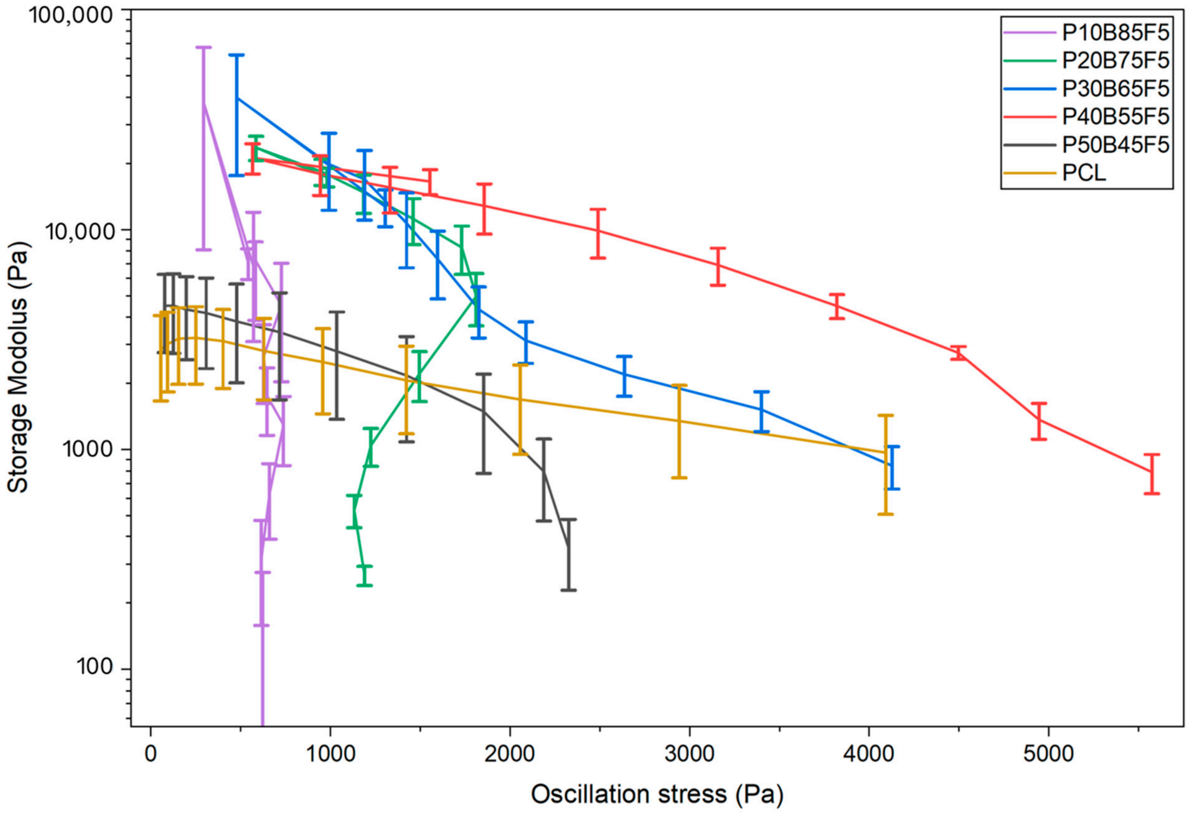

Figure S4: Storage modulus (G’) of all formulated ink compositions as a function of oscillation stress, showing the stress-dependent viscoelastic response and the onset of structural softening across compositions, with data being presented as mean ± standard error.

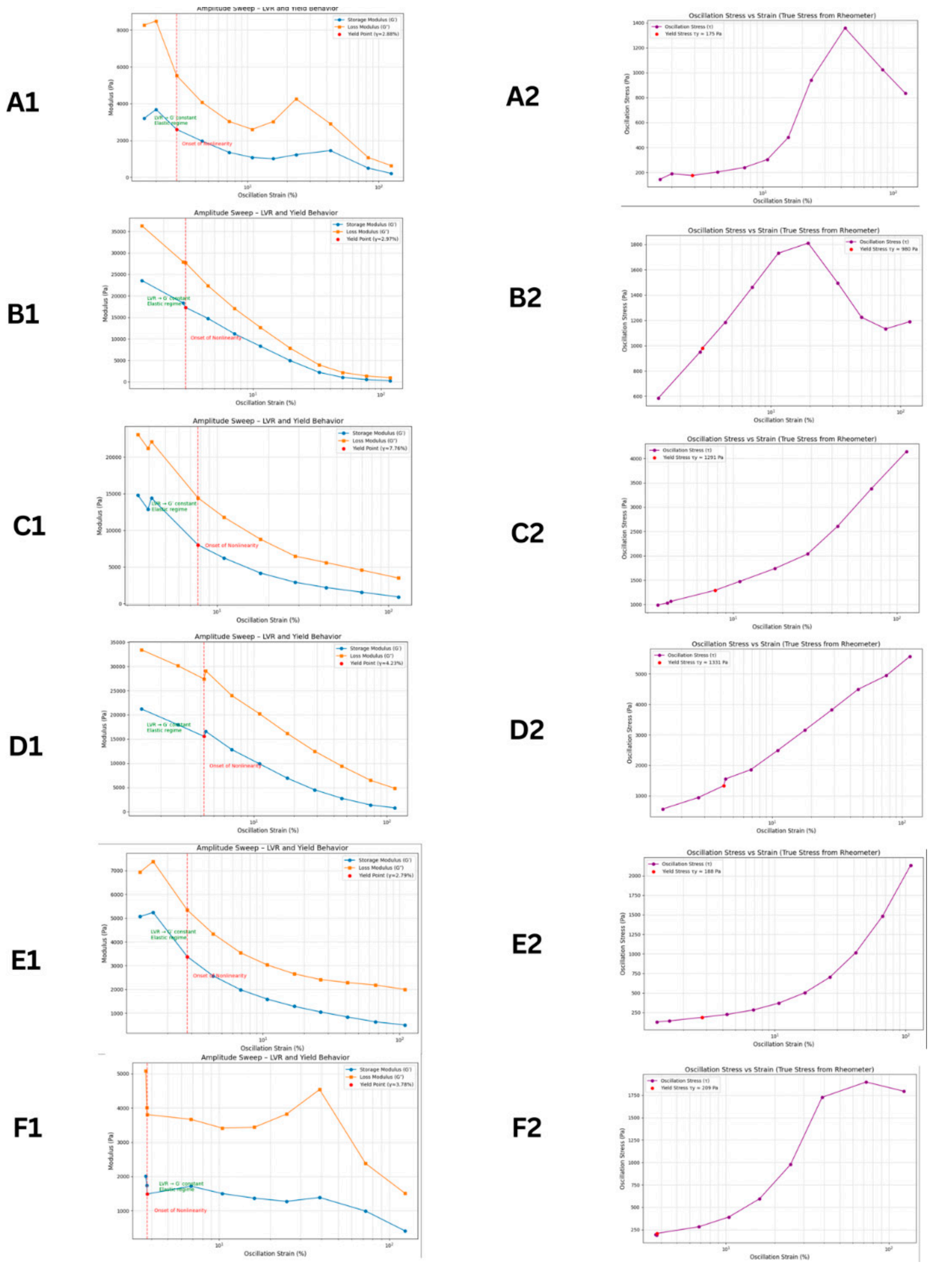

Figure S5: Amplitude sweep and yield behavior of composite scaffold inks. Panels A1–F1 show the amplitude sweep results identifying the linear viscoelastic region (LVR), G', loss modulus (G"), and the onset of nonlinearity, with yield points marked by dashed vertical lines. Panels A2–F2 present the corresponding oscillatory (true) stress as a function of oscillatory strain, with yield stress indicated for each formulation. A-P10B85F5, B-P20B75F5, C-P30B65F5, D-P40B55F5, E-P50B45F5 and F-PCL. Data are presented as mean values from three independent measurements (n = 3).

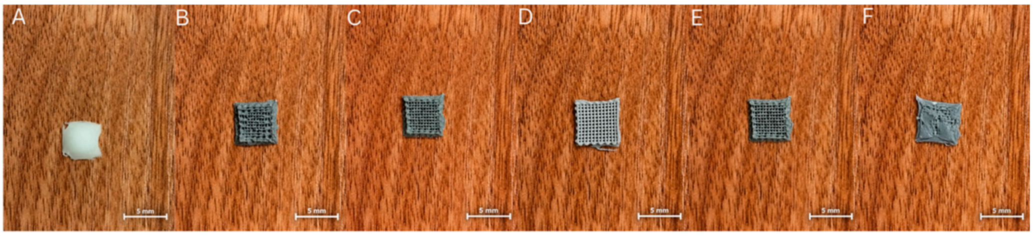

Figure S6: Representative images of scaffolds fabricated by direct ink writing using a 0.51 mm inner-diameter nozzle: (A) PCL, (B) P10B85F5, (C) P20B75F5, (D) P30B65F5, (E) P40B55F5, and (F) P50B45F5.

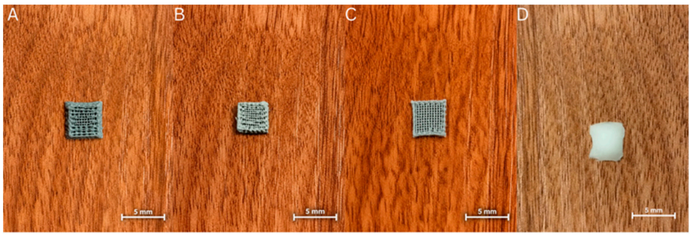

Figure S7: Representative images of scaffolds fabricated by direct ink writing using a 0.41 mm inner-diameter nozzle: (A) P10B85F5, (B) P20B75F5, (C) P30B65F5 and (D) PCL.

Table S1. One-way ANOVA of rheological parameters for PCL and PCL/β-TCP/Fe_3O_4 formulations (n = 3 per group).

Parameter	F (5,12)	p-value	Significance
Storage modulus, G′	12.014	0.000248	***
Loss modulus, G″	18.496	0.0000288	****
Oscillation strain (%)	2.716	0.0725	ns
Oscillation stress (Pa)	8.878	0.001007	**

Reported p-values are adjusted for multiple comparisons. Statistical significance is indicated as $p < 0.05$ (*), $p < 0.01$ (**), $p < 0.001$ (***), and $p < 0.0001$ (****); ns indicates $p \geq 0.05$.

Table S2. Tukey HSD pairwise comparisons. Statistical significance is indicated as $p < 0.05$ (*), $p < 0.01$ (**), $p < 0.001$ (***), and $p < 0.0001$ (****); ns indicates $p \geq 0.05$.

Parameter	Comparison	Tukey-adjusted p	Significance
**Storage modulus**	P10B85F5–P20B75F5	0.0017	**
	P10B85F5–P30B65F5	0.4728	ns
	P10B85F5–P40B55F5	0.0083	**
	P10B85F5–P50B45F5	0.9998	ns
	P10B85F5–PCL	0.9987	ns
	P20B75F5–P30B65F5	0.0371	*

Parameter	Comparison	Tukey-adjusted p	Significance
	P20B75F5–P40B55F5	0.9225	ns
	P20B75F5–P50B45F5	0.0027	**
	P20B75F5–PCL	0.0010	**
	P30B65F5–P40B55F5	0.1764	ns
	P30B65F5–P50B45F5	0.6225	ns
	P30B65F5–PCL	0.2918	ns
	P40B55F5–P50B45F5	0.0129	*
	P40B55F5–PCL	0.0044	**
	P50B45F5–PCL	0.9846	ns
**Loss modulus**	P10B85F5–P20B75F5	0.0006	***
	P10B85F5–P30B65F5	0.2233	ns
	P10B85F5–P40B55F5	0.0007	***
	P10B85F5–P50B45F5	1.0000	ns
	P10B85F5–PCL	0.9964	ns
	P20B75F5–P30B65F5	0.0295	*
	P20B75F5–P40B55F5	1.0000	ns
	P20B75F5–P50B45F5	0.0005	***
	P20B75F5–PCL	0.0003	***
	P30B65F5–P40B55F5	0.0372	*
	P30B65F5–P50B45F5	0.2066	ns
	P30B65F5–PCL	0.1083	ns
	P40B55F5–P50B45F5	0.0007	***
	P40B55F5–PCL	0.0004	***
	P50B45F5–PCL	0.9980	ns
**Oscillation strain**	P10B85F5–P20B75F5	1.0000	ns
	P10B85F5–P30B65F5	0.0942	ns
	P10B85F5–P40B55F5	0.9566	ns
	P10B85F5–P50B45F5	1.0000	ns
	P10B85F5–PCL	0.9927	ns
	P20B75F5–P30B65F5	0.0914	ns
	P20B75F5–P40B55F5	0.9526	ns
	P20B75F5–P50B45F5	1.0000	ns
	P20B75F5–PCL	0.9915	ns
	P30B65F5–P40B55F5	0.3266	ns
	P30B65F5–P50B45F5	0.0865	ns

Parameter	Comparison	Tukey-adjusted p	Significance
	P30B65F5–PCL	0.2209	ns
	P40B55F5–P50B45F5	0.9444	ns
	P40B55F5–PCL	0.9997	ns
	P50B45F5–PCL	0.9890	ns
**Oscillation stress**	P10B85F5–P20B75F5	0.1070	ns
	P10B85F5–P30B65F5	0.0127	*
	P10B85F5–P40B55F5	0.0099	**
	P10B85F5–P50B45F5	1.0000	ns
	P10B85F5–PCL	1.0000	ns
	P20B75F5–P30B65F5	0.7937	ns
	P20B75F5–P40B55F5	0.7146	ns
	P20B75F5–P50B45F5	0.1151	ns
	P20B75F5–PCL	0.1305	ns
	P30B65F5–P40B55F5	1.0000	ns
	P30B65F5–P50B45F5	0.0138	*
	P30B65F5–PCL	0.0157	*
	P40B55F5–P50B45F5	0.0107	*
	P40B55F5–PCL	0.0122	*
	P50B45F5–PCL	1.0000	ns

Script S1: Particle analysis

For calibration code (scale bar)

```
import cv2
import numpy as np
import matplotlib.pyplot as plt

filename = "btcp1.4.tif"
scale_bar_um = 30.0

img = cv2.imread(filename, cv2.IMREAD_GRAYSCALE)

if img is None:
    raise FileNotFoundError(f"Could not find {filename}")

h, w = img.shape

# Bottom 25% of image
bottom = img[int(h * 0.75):h, :]

# Threshold
_, binary = cv2.threshold(
    bottom, 0, 255,
    cv2.THRESH_BINARY + cv2.THRESH_OTSU
)

# Connected components
num_labels, labels, stats, centroids = cv2.connectedComponentsWithStats(
    binary, connectivity=8
)

candidates = []

for i in range(1, num_labels):

    x = stats[i, cv2.CC_STAT_LEFT]
    y = stats[i, cv2.CC_STAT_TOP]
    width = stats[i, cv2.CC_STAT_WIDTH]
    height = stats[i, cv2.CC_STAT_HEIGHT]
    area = stats[i, cv2.CC_STAT_AREA]

    aspect_ratio = width / max(height, 1)

    if width >= 20 and height <= 20 and aspect_ratio >= 3:
```

```
        candidates.append(
        (i, x, y, width, height, area, aspect_ratio)
        )

print("Possible scale-bar candidates:\n")

for j, c in enumerate(candidates):
        print(
        f"Candidate {j}: "
        f"x={c[1]}, y={c[2]}, "
        f"width={c[3]} pixels, "
        f"height={c[4]} pixels"
        )

# Show candidates
display_img = cv2.cvtColor(bottom, cv2.COLOR_GRAY2RGB)

for j, c in enumerate(candidates):

        _, x, y, width, height, _, _ = c

        cv2.rectangle(
        display_img,
        (x, y),
        (x + width, y + height),
        (255, 0, 0),
        2
        )

        cv2.putText(
        display_img,
        str(j),
        (x, max(y - 5, 10)),
        cv2.FONT_HERSHEY_SIMPLEX,
        0.5,
        (255, 0, 0),
        1
        )

plt.figure(figsize=(16, 5))
plt.imshow(display_img)
plt.title(f"{filename}: identify the box around the 30 µm scale bar")
plt.axis("off")
plt.show()
```

```
scale_bar_pixels =  # ENTER THE CORRECT WIDTH HERE

calibration = scale_bar_um / scale_bar_pixels

print(f"Calibration = {calibration:.6f} µm/pixel")

# ==========================================================
# STEP 1 — IMPORT LIBRARIES AND DEFINE ANALYSIS SETTINGS
# ==========================================================

import cv2
import numpy as np
import pandas as pd
import matplotlib.pyplot as plt

from scipy import ndimage as ndi

from skimage import (
    filters,
    morphology,
    measure,
    segmentation,
    feature
)

# ----------------------------------------------------------
# VERIFIED SCALE-BAR CALIBRATIONS
# ----------------------------------------------------------

image_info = {
    "bTCP1.2.tif": 0.416667,   # VERIFY whether scale bar = 72 px
    "btcp1.4.tif": 0.384615,
    "btcp1.5.tif": 0.384615,
    "btcp2.1.tif": 0.384615,
    "btcp2.2.tif": 0.298507
}

# Watershed parameter selected by visual inspection
MIN_DISTANCE = 7

# Minimum projected area used to remove very small
# segmented regions/noise
MIN_AREA_UM2 = 3.47

print("Analysis settings loaded.")
```

```
for filename, calibration in image_info.items():
    min_pixels = int(
    np.ceil(MIN_AREA_UM2 / calibration**2)
    )

    print(
    f"{filename}: "
    f"{calibration:.6f} µm/pixel | "
    f"minimum area = {min_pixels} pixels²"
    )

# ============================================================
# STEP 2 — SEGMENT PARTICLES AND CALCULATE ECD
# ============================================================

all_results = []

for filename, um_per_pixel in image_info.items():

    print(f"\nProcessing {filename}...")

    # Load grayscale SEM image
    img = cv2.imread(
    filename,
    cv2.IMREAD_GRAYSCALE
    )

    if img is None:
    print(f"WARNING: Could not find {filename}. Skipping.")
    continue

    # --------------------------------------------------------
    # Remove SEM information/footer region
    # --------------------------------------------------------

    crop_height = 650
    micrograph = img[:crop_height, :]

    # --------------------------------------------------------
    # Image preprocessing
    # --------------------------------------------------------

    blurred = cv2.GaussianBlur(
    micrograph,
    (5, 5),
    0
```

```
)

clahe = cv2.createCLAHE(
clipLimit=2.0,
tileGridSize=(8, 8)
)

enhanced = clahe.apply(blurred)

# ------------------------------------------------------
# Otsu thresholding
# ------------------------------------------------------

threshold = filters.threshold_otsu(enhanced)

binary = enhanced > threshold

# ------------------------------------------------------
# Convert physical area threshold to pixels
# ------------------------------------------------------

min_size_pixels = int(
np.ceil(
MIN_AREA_UM2 /
(um_per_pixel ** 2)
)
)

print(
f"Calibration = {um_per_pixel:.6f} µm/pixel"
)

print(
f"Minimum area threshold = "
f"{min_size_pixels} pixels²"
)

# ------------------------------------------------------
# Morphological cleaning
# ------------------------------------------------------

binary_clean = morphology.remove_small_objects(
binary,
min_size=min_size_pixels
)
```

```
binary_clean = morphology.remove_small_holes(
binary_clean,
area_threshold=min_size_pixels
)

# -------------------------------------------------------
# Distance transform
# -------------------------------------------------------

distance = ndi.distance_transform_edt(
binary_clean
)

# -------------------------------------------------------
# Identify particle centers
# -------------------------------------------------------

coords = feature.peak_local_max(
distance,
min_distance=MIN_DISTANCE,
labels=binary_clean
)

marker_mask = np.zeros(
distance.shape,
dtype=bool
)

marker_mask[tuple(coords.T)] = True

markers, _ = ndi.label(marker_mask)

# -------------------------------------------------------
# Watershed segmentation
# -------------------------------------------------------

labels = segmentation.watershed(
-distance,
markers,
mask=binary_clean
)

# Remove particles touching image borders
labels_clean = segmentation.clear_border(
labels
)
```

```
# ------------------------------------------------------
# Measure segmented objects
# ------------------------------------------------------

props = measure.regionprops(labels_clean)

retained = 0

for p in props:

area_px = p.area

area_um2 = (
area_px *
um_per_pixel**2
)

# Apply final physical-area criterion
if area_um2 < MIN_AREA_UM2:
continue

# Equivalent circular diameter
ecd_um = 2 * np.sqrt(
area_um2 / np.pi
)

# Maximum Feret diameter
feret_um = (
p.feret_diameter_max *
um_per_pixel
)

all_results.append({

"Image": filename,

"Particle_ID": p.label,

"Area_pixels": area_px,

"Area_um2": area_um2,

"ECD_um": ecd_um,

"Feret_um": feret_um,
```

```
        "Solidity": p.solidity,

        "Eccentricity": p.eccentricity,

        "Centroid_Y": p.centroid[0],

        "Centroid_X": p.centroid[1]

        })

        retained += 1

        print(
        f"Retained segmented features = {retained}"
        )

# ----------------------------------------------------------
# Create master dataframe
# ----------------------------------------------------------

data = pd.DataFrame(all_results)

print("\n====================================")
print("ANALYSIS COMPLETE")
print("====================================")

print(
        f"Total retained segmented features = {len(data)}"
)

print("\nFeatures per SEM image:")

print(
        data["Image"].value_counts()
)

display(data.head(10))

Processing btcp1.4.tif...
Calibration = 0.384615 µm/pixel
Minimum area threshold = 24 pixels²
Retained segmented features = 1127

Processing btcp1.5.tif...
Calibration = 0.384615 µm/pixel
```

Minimum area threshold = 24 pixels²
Retained segmented features = 1080

Processing btcp2.1.tif...
Calibration = 0.384615 µm/pixel
Minimum area threshold = 24 pixels²
Retained segmented features = 1276

Processing btcp2.2.tif...
Calibration = 0.298507 µm/pixel
Minimum area threshold = 39 pixels²
Retained segmented features = 989

====================================
ANALYSIS COMPLETE
====================================
Total retained segmented features = 5781
Features per SEM image:
Image
bTCP1.2.tif 1309
btcp2.1.tif 1276
btcp1.4.tif 1127
btcp1.5.tif 1080
btcp2.2.tif 989
Name: count, dtype: int64

	Image	Particle_ID	Area_pixels	Area_um2	ECD_um	Feret_um	Solidity	Eccentricity	Centroid_Y	Centroid_X
0	bTCP1.2.tif	3	231.0	40.104231	7.145788	9.762820	0.891892	0.750145	15.536797	70.748918
1	bTCP1.2.tif	4	75.0	13.020854	4.071691	5.416671	0.797872	0.551923	6.773333	121.373333
2	bTCP1.2.tif	11	41.0	7.118067	3.010482	3.560004	1.000000	0.635848	9.121951	608.439024
3	bTCP1.2.tif	12	37.0	6.423621	2.859862	4.658479	0.948718	0.928081	11.270270	622.432432
4	bTCP1.2.tif	14	417.0	72.395949	9.600905	15.166908	0.727749	0.869815	18.194245	665.340528
5	bTCP1.2.tif	17	81.0	14.062523	4.231425	5.153886	0.910112	0.487031	7.185185	986.370370

**6**	bTCP 1.2.tif	22	69.0	11.979186	3.905429	5.270467	0.784091	0.402193	9.362319	576.420290
**7**	bTCP 1.2.tif	23	130.0	22.569481	5.360630	7.288696	0.896552	0.716477	13.923077	789.553846
**8**	bTCP 1.2.tif	24	65.0	11.284740	3.790538	5.068973	0.878378	0.775431	10.646154	852.538462
**9**	bTCP 1.2.tif	27	116.0	20.138921	5.063761	7.120009	0.816901	0.776195	16.594828	225.077586

```
# ==========================================================
# STEP 3 — PARTICLE-SIZE STATISTICS
# ==========================================================

per_image = data.groupby("Image")["ECD_um"].agg(
        n="count",
        Mean_ECD_um="mean",
        SD_um="std",
        Median_ECD_um="median",
        Min_ECD_um="min",
        Max_ECD_um="max"
)

per_image["P10_um"] = (
    data.groupby("Image")["ECD_um"]
        .quantile(0.10)
)

per_image["P50_um"] = (
    data.groupby("Image")["ECD_um"]
        .quantile(0.50)
)

per_image["P90_um"] = (
    data.groupby("Image")["ECD_um"]
        .quantile(0.90)
)

print("\nPER-IMAGE RESULTS")

display(
        per_image.round(2)
)
```

```
# ------------------------------------------------------------
# Pooled statistics
# ------------------------------------------------------------

sizes = data["ECD_um"].dropna()

print("\nFINAL POOLED β-TCP SEM PARTICLE-SIZE ANALYSIS")
print("------------------------------------------------")

print(
    f"Number of segmented features = {len(sizes)}"
)

print(
    f"Mean ECD = "
    f"{sizes.mean():.2f} ± "
    f"{sizes.std(ddof=1):.2f} µm"
)

print(
    f"Median ECD = "
    f"{sizes.median():.2f} µm"
)

print(
    f"P10 = "
    f"{sizes.quantile(0.10):.2f} µm"
)

print(
    f"P50 = "
    f"{sizes.quantile(0.50):.2f} µm"
)

print(
    f"P90 = "
    f"{sizes.quantile(0.90):.2f} µm"
)

print(
    f"Observed range = "
    f"{sizes.min():.2f}–"
    f"{sizes.max():.2f} µm"
)
```

PER-IMAGE RESULTS

Image	n	Mean_ECD_um	SD_um	Median_ECD_um	Min_ECD_um	Max_ECD_um	P10_um	P50_um	P90_um
**bTCP 1.2.tif**	1309	6.54	3.05	5.93	2.10	25.50	3.32	5.93	10.30
**btcp1.4.tif**	1127	6.28	3.28	5.59	2.13	29.11	2.99	5.59	10.52
**btcp1.5.tif**	1080	6.25	3.59	5.33	2.13	33.14	2.94	5.33	10.13
**btcp2.1.tif**	1276	6.09	2.79	5.49	2.13	19.16	3.01	5.49	10.10
**btcp2.2.tif**	989	5.30	2.49	4.75	2.10	15.28	2.67	4.75	8.94

```
FINAL POOLED β-TCP SEM PARTICLE-SIZE ANALYSIS
----------------------------------------------
Number of segmented features = 5781
Mean ECD = 6.12 ± 3.09 μm
Median ECD = 5.46 μm
P10 = 2.94 μm
P50 = 5.46 μm
P90 = 10.05 μm
Observed range = 2.10–33.14 μm
```

```
# ==========================================================
# STEP 4 — FINAL SEGMENTATION QC
# ==========================================================

fig, axes = plt.subplots(
        len(image_info),
        1,
        figsize=(12, 30)
)

for ax, (filename, um_per_pixel) in zip(
        axes,
        image_info.items()
):

        img = cv2.imread(
        filename,
        cv2.IMREAD_GRAYSCALE
```

```
)

crop_height = 650
micrograph = img[:crop_height, :]

blurred = cv2.GaussianBlur(
micrograph,
(5, 5),
0
)

clahe = cv2.createCLAHE(
clipLimit=2.0,
tileGridSize=(8, 8)
)

enhanced = clahe.apply(blurred)

threshold = filters.threshold_otsu(
enhanced
)

binary = enhanced > threshold

min_size_pixels = int(
np.ceil(
MIN_AREA_UM2 /
um_per_pixel**2
)
)

binary_clean = morphology.remove_small_objects(
binary,
min_size=min_size_pixels
)

binary_clean = morphology.remove_small_holes(
binary_clean,
area_threshold=min_size_pixels
)

distance = ndi.distance_transform_edt(
binary_clean
)

coords = feature.peak_local_max(
```

```
distance,
min_distance=MIN_DISTANCE,
labels=binary_clean
)

marker_mask = np.zeros(
distance.shape,
dtype=bool
)

marker_mask[tuple(coords.T)] = True

markers, _ = ndi.label(marker_mask)

labels = segmentation.watershed(
-distance,
markers,
mask=binary_clean
)

labels_clean = segmentation.clear_border(
labels
)

# -------------------------------------------------------
# Remove watershed objects below physical threshold
# -------------------------------------------------------

valid_labels = []

for region in measure.regionprops(labels_clean):

area_um2 = (
region.area *
um_per_pixel**2
)

if area_um2 >= MIN_AREA_UM2:
valid_labels.append(region.label)

final_labels = np.where(
np.isin(labels_clean, valid_labels),
labels_clean,
0
)
```

```
        boundaries = segmentation.find_boundaries(
        final_labels,
        mode="outer"
        )

        ax.imshow(
        micrograph,
        cmap="gray"
        )

        overlay = np.ma.masked_where(
        ~boundaries,
        boundaries
        )

        ax.imshow(
        overlay,
        cmap="autumn",
        alpha=0.9
        )

        ax.set_title(
        f"{filename} | "
        f"Retained features = {len(valid_labels)} | "
        f"min_distance = {MIN_DISTANCE}"
        )

        ax.axis("off")

plt.tight_layout()

plt.savefig(
    "beta_TCP_all_segmentation_QC.png",
        dpi=300,
        bbox_inches="tight"
)

plt.show()

# ============================================================
# STEP 5 — FINAL β-TCP PARTICLE-SIZE DISTRIBUTION
# ============================================================

sizes = data["ECD_um"].dropna()

plt.figure(figsize=(7, 5))
```

```
weights = (
    np.ones(len(sizes)) *
    100 / len(sizes)
)

plt.hist(
    sizes,
    bins=np.arange(2, 36, 2),
    weights=weights,
    edgecolor="black",
    linewidth=1.0
)

plt.axvline(
    sizes.mean(),
    linestyle="--",
    linewidth=1.5,
    label=f"Mean = {sizes.mean():.2f} µm"
)

plt.axvline(
    sizes.median(),
    linestyle=":",
    linewidth=1.5,
    label=f"Median = {sizes.median():.2f} µm"
)

plt.xlabel(
    "Equivalent circular diameter (µm)",
    fontsize=12
)

plt.ylabel(
    "Relative frequency (%)",
    fontsize=12
)

plt.xlim(0, 35)

plt.legend(
    frameon=False,
    fontsize=10
)

plt.tight_layout()
```

```
plt.savefig(
    "beta_TCP_final_particle_size_distribution.png",
          dpi=600,
          bbox_inches="tight"
)

plt.show()
```
